# SP1 antagonizes H3K27me3 to shape chromatin landscapes for RNA polymerase II recruitment during gastrulation

**DOI:** 10.1093/nar/gkag305

**Published:** 2026-04-13

**Authors:** Xipeng Shen, Yuting Wen, Xiaohan Tang, Yujiao Liu, Wensi Li, Wenhao Chen, Shuheng Yang, Lidan Wang, Haibo Yang, Kunyan Liu, Lei Li, Yunlong Xiang

**Affiliations:** Department of Rheumatology & Immunology Children’s Hospital of Chongqing Medical University, National Clinical Research Center for Children and Adolescents' Health and Diseases, Ministry of Education Key Laboratory of Child Development and Disorders. Chongqing Key Laboratory of Child Rare Diseases in Infection and Immunity, School of Basic Medical Sciences, Chongqing, 400010, China; Center for Medical Epigenetics, School of Basic Medical Sciences, Chongqing Medical University, Chongqing, 400010, China; Department of Rheumatology & Immunology Children’s Hospital of Chongqing Medical University, National Clinical Research Center for Children and Adolescents' Health and Diseases, Ministry of Education Key Laboratory of Child Development and Disorders. Chongqing Key Laboratory of Child Rare Diseases in Infection and Immunity, School of Basic Medical Sciences, Chongqing, 400010, China; Center for Medical Epigenetics, School of Basic Medical Sciences, Chongqing Medical University, Chongqing, 400010, China; Department of Reproductive Medicine Center, The First Affiliated Hospital of Chongqing Medical University, Chongqing, 400010, China; Center for Medical Epigenetics, School of Basic Medical Sciences, Chongqing Medical University, Chongqing, 400010, China; Center for Medical Epigenetics, School of Basic Medical Sciences, Chongqing Medical University, Chongqing, 400010, China; Center for Medical Epigenetics, School of Basic Medical Sciences, Chongqing Medical University, Chongqing, 400010, China; Center for Medical Epigenetics, School of Basic Medical Sciences, Chongqing Medical University, Chongqing, 400010, China; Center for Medical Epigenetics, School of Basic Medical Sciences, Chongqing Medical University, Chongqing, 400010, China; Information Center of Chongqing Medical University, Chongqing, 400010, China; Center for Medical Epigenetics, School of Basic Medical Sciences, Chongqing Medical University, Chongqing, 400010, China; State Key Laboratory of Stem Cell and Reproductive Biology, Key Laboratory of Organ Regeneration and Reconstruction, UCAS/IOZ/CAS, Beijing, 100101, China; Beijing Institute of Stem Cell and Regenerative Medicine, Beijing, 100100, China; Department of Rheumatology & Immunology Children’s Hospital of Chongqing Medical University, National Clinical Research Center for Children and Adolescents' Health and Diseases, Ministry of Education Key Laboratory of Child Development and Disorders. Chongqing Key Laboratory of Child Rare Diseases in Infection and Immunity, School of Basic Medical Sciences, Chongqing, 400010, China; Center for Medical Epigenetics, School of Basic Medical Sciences, Chongqing Medical University, Chongqing, 400010, China; Department of Reproductive Medicine Center, The First Affiliated Hospital of Chongqing Medical University, Chongqing, 400010, China

## Abstract

Following implantation, the epiblast undergoes gastrulation to form the three germ layers, a process requiring precise temporal control of developmental gene expression. However, the mechanisms governing RNA polymerase II (Pol II) engagement at developmental gene promoters during this critical stage remain poorly understood. Here, we present a genome-wide analysis of Pol II occupancy in mouse post-implantation embryos, revealing that nearly half of bivalent promoters are bound by Pol II in a lineage-specific and temporally ordered manner. This recruitment follows a stepwise chromatin remodeling cascade, with initial deposition of H3K27me3, followed by H3K4me3 acquisition and Pol II engagement. Through genetic perturbation, we show that KMT2B promotes Pol II loading via H3K4me3 deposition, whereas the Polycomb component EED restricts this process by maintaining H3K27me3. Notably, we identify the transcription factor SP1 as a critical facilitator of Pol II recruitment at bivalent loci. SP1 binding coincides with reduced H3K27me3 levels and enhanced Pol II occupancy, and its loss leads to chromatin re-silencing and transcriptional failure. Together, our findings establish a chromatin-based regulatory framework in which SP1 and histone modifications cooperatively license the transcriptional activation of developmental genes during germ layer formation.

## Graphical Abstract

**Graphical Abstract:**
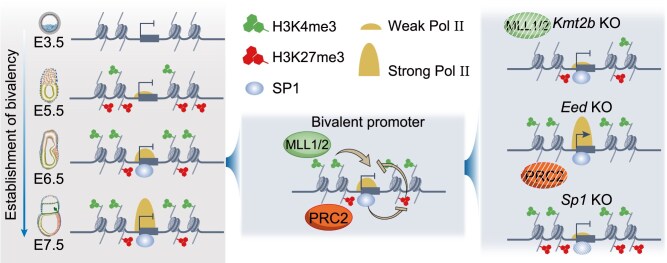

## Introduction

The transformation of a single-cell zygote into a multicellular organism with complex tissues and organs is driven by coordinated cell fate decisions. Gastrulation marks a pivotal developmental window during which the epiblast undergoes extensive remodeling to generate the three germ layers—the cellular foundation of the embryo proper. This process is tightly regulated by a complex network of transcriptional and epigenetic programs [1–3]. Yet, how transcriptional activity is precisely initiated and regulated at developmental genes during this stage remains poorly understood.

A defining feature of developmental gene regulation in mammalian cells is known as “bivalency”. This state is characterized by the simultaneous presence of two opposing histone modifications at promoters: trimethylated histone H3 Lys4 (H3K4me3), associated with transcriptional activation, and trimethylated histone H3 Lys27 (H3K27me3), linked to transcriptional repression [4–8]. Bivalent chromatin is conserved across species and enriched at key transcription factor loci such as HOX, PAX, FOX, SOX, POU, FGF, and WNT [9–12]. The bivalent state is dynamically reconfigured during early mouse embryogenesis. Maternal non-canonical H3K4me3 is rapidly converted to the canonical form after fertilization, while H3K27me3 persists until the blastocyst stage, with diminished enrichment at developmental gene promoters such as the *Hox* cluster, resulting in a transient loss of bivalency [13–16]. It is re-established in post-implantation epiblast by E6.5, with both H3K4me3 and H3K27me3 showing a broader and stronger distribution at developmental gene promoters [17]. Genetic studies have shown that zygotic knockout of histone-lysine N-methyltransferase 2B (KMT2B, also known as MLL2), which is a major enzyme catalyzing the H3K4me3 modification on bivalent genes, results in delayed embryonic development and lethality by embryonic day 11.5 (E11.5) [18]. Loss of embryonic ectoderm development protein (EED), a core component of the Polycomb repressive complex 2 (PRC2), leads to a significant reduction in H3K27me3 and embryonic lethality by E8.5 [19, 20]. These findings underscore the essential role of bivalency in regulating developmental gene expression and ensuring normal embryonic development.

Bivalent chromatin is thought to maintain developmental genes in a transcriptionally “poised” or “primed” state, enabling rapid activation in response to differentiation cues. At these promoters, RNA polymerase II (Pol II) is preloaded but transcriptionally inactive, lacking Ser2 phosphorylation (Ser2p) associated with productive elongation [21–23]. This configuration allows transcription to proceed through the first exon–exon junction but fails to generate full-length transcripts, distinguishing it from classical promoter-proximal pausing, which yields only short RNAs of 10–50 nucleotides [24, 25]. Pol II is detected at only ∼10–30% of bivalent promoters in early Drosophila embryos and mouse embryonic stem cells [22, 26]. Following fertilization, Pol II is gradually re-engaged through sequential loading and configuration steps to overcome zygotic transcriptional quiescence [27], but this primarily supports housekeeping gene expression. In contrast, developmental gene promoters remain largely unoccupied by Pol II at the blastocyst stage, coinciding with the temporary loss of bivalency. Consequently, both bivalent chromatin and Pol II occupancy must be re-established during post-implantation development to permit timely activation of lineage regulators [16, 17].

The regulatory logic guiding Pol II recruitment to bivalent promoters is complex and remains a subject of debate. While PRC2-mediated H3K27me3 is generally considered repressive, studies have shown that developmental genes may remain inactive even after PRC2 loss [4, 28, 29]. JARID2, a component of PRC2, is required for the recruitment of Pol II to developmental regulators in mouse ESCs [30]. Conversely, H3K4me3 has been implicated in transcriptional initiation and Pol II loading, yet its absence appears to affect elongation dynamics rather than Pol II recruitment per se [31]. Although transcription factors are proposed to facilitate Pol II recruitment to developmental promoters, the identity of such factors during germ layer formation remains elusive. Moreover, most existing studies have focused on ESCs or preimplantation stages, leaving a critical gap in our understanding of how Pol II is engaged at developmental genes during post-implantation embryogenesis.

Here, we show that Pol II recruitment to developmental gene promoters during gastrulation is orchestrated by the antagonistic activities of H3K4me3 and H3K27me3. Importantly, we identify the transcription factor SP1 as a key regulator that facilitates Pol II binding by counteracting PRC2-mediated H3K27me3 deposition. Disruption of this chromatin balance compromises Pol II recruitment and causes aberrant activation of lineage-specifying genes, ultimately resulting in embryonic lethality. These findings reveal how chromatin modifications and transcription factors collaboratively govern transcriptional activation during early mammalian development.

## Materials and methods

### Cell culture

HeLa cells [American Type Culture Collection (ATCC), Manassas, VA, USA] were cultured in DMEM medium supplemented with 10% fetal bovine serum at 37°C, 95% humidity, and 5% CO_2_.

### Separation of germ layers

C57BL/6N female mice (4–8 weeks old) were purchased from Beijing Vital River Laboratory Animal Technology Co., Ltd. All animals were maintained in accordance with the guidelines of the Animal Care and Use Committee of Chongqing Medical University. Germ layer separation during the gastrulation stage was performed as previously described [32]. Briefly, adult PWK/PhJ male mice (8–10 weeks old) were mated with C57BL/6N females (4–8 weeks old). The presence of a vaginal plug on the following day was considered as embryonic day 0.5 (E0.5). Pregnant females were euthanized by cervical dislocation at E6.5. Embryos were carefully dissected to remove the decidual and Reichert’s membranes. Using silicon glass needles, the extraembryonic and embryonic regions were meticulously separated in M2 medium (Sigma). Embryos were then washed in serum-free DMEM and enzymatically dissociated by a 10-min incubation in a pancreatin/trypsin solution on ice. The embryonic region was carefully aspirated into a silicon glass tube to facilitate the separation of the visceral endoderm (VE) and epiblast (Epi). The extraembryonic ectoderm (ExE) and extraembryonic visceral endoderm (ExVE) were also separated as described. For E7.5 embryos, the same methods were employed to obtain the endoderm layer (End), ExE, and ExVE. The mesoderm (Mes) was gently dissected from the remaining embryonic tissue, which resembled a pair of wings attached to the ‘J-shaped’ primitive streak. The ectoderm layer (Ect) was obtained by excising the primitive streak.

### Generating gene knockout embryos

CRISPR-ZEN technology was employed to generate constitutive knockout embryos by introducing Cas9 and sgRNA complexes directly into mouse zygotes [33]. Female C57BL/6N mice (5–6 weeks old) were intraperitoneally injected with Pregnant Mare Serum Gonadotropin (PMSG) (10 IU), followed by Human Chorionic Gonadotropin (HCG) (10 IU) 46–48 h later. After mating with PWK/PHJ male mice (8–10 weeks old), females exhibiting a vaginal plug were sacrificed the next morning to obtain PN3-PN4 zygotes. The Cumulus-Oocyte Complexes (COCs) were treated with 0.3 mg/ml hyaluronidase (Sigma) to remove cumulus cells. Zygotes were then transferred to Acidic Tyrode’s solution (Sigma) for 20 s to thin the zona pellucida. Meanwhile, the Cas9 protein (IDT) was incubated with sgRNAs (Supplementary Table Sheet 1) in electroporation buffer at 37°C for 15 min. Zygotes were then transferred to Opti-MEM and mixed with the sgRNA-Cas9 solution. Electroporation was performed using the following parameters: 5 pulses lasting 0.3 ms at 30 V with a 0.1 ms interval using the Gene Pulser Xcell (Bio-Rad). Embryos were then cultured in KSOM + AA (Thermo Fisher Scientific) at 37°C under 5% CO_2_ until the two-cell stage and subsequently transferred to E0.5 pseudopregnancy foster mothers.

### Genotyping of knockout embryos

The ExVE from each embryo was collected for genotyping. The ExVE was washed in M2 medium, and excess liquid was carefully removed. Subsequently, 10 µl of lysis buffer containing 0.2 µg/µl proteinase K (Roche) was added to facilitate the release of genomic DNA. The mixture was incubated at 55°C for 60 min, followed by a 10-min denaturation step at 95°C to inactivate the proteinase K. PCR reaction was then performed for genotyping using the primers listed in Supplementary Table Sheet 2. Embryos with deletions in both alleles of the target gene were considered as knockouts, while those without allele deletions were collected as controls.

### H&E staining of the embryo

For histological analysis, embryos were fixed in 4% paraformaldehyde at 4°C overnight, dehydrated through an ethanol gradient, embedded in paraffin, and sectioned at 5 μm thickness. Sections were stained with hematoxylin and eosin according to standard procedures and imaged using a brightfield microscope.

### RNA-seq library construction

The RNA-seq library was generated following the Smart-seq2 protocol [34]. Samples were permeabilized in 5 µl lysis buffer containing 0.2% Triton X-100 for 30 min on ice. After centrifugation, 2 µl of the supernatant was transferred to a pre-cooled tube and reverse transcribed into cDNA. The library construction was then performed using the One-step DNA Lib Prep Kit for Illumina V2 (Abclonal) according to the manufacturer’s instructions.

### ChIP-seq library construction

ChIP-seq was performed with minor modifications to the STAR ChIP-seq protocol [13]. Samples were lysed in a buffer containing 0.5% Tween-20, 0.5% NP-40, and 0.1% SDS, with gentle pipetting to release genomic DNA. The mixture was then digested with micrococcal nuclease (MNase) (Sigma) at 37°C for 5 min. Primary antibodies—anti-Pol II RBP1 (BioLegend, cat: 664 906; lot: B33493), anti-Phospho-POLR2A CTD-S2 (Abclonal, cat: AP0749; lot: 21 155 550 101), anti-SP1 (Proteintech, cat: 21962–1-AP; lot: 77 360), anti-H3K4me3 (Millipore, cat: 04–745; lot: 3 689 889) or anti-H3K27me3 (Abclonal, cat: A2363; lot: 5 500 016 460)—were added, followed by overnight incubation at 4°C. Protein A Dynabeads (Thermo Fisher Scientific) were subsequently added to the solution and incubated for 3–4 h. The beads were washed five times with RIPA buffer and once with LiCl wash buffer. DNA fragments were eluted by proteinase K treatment. The eluted DNA fragments were subjected to library construction using the NEB Next® Ultra II DNA Library Prep Kit, following the manufacturer’s instructions. Both ChIP-seq and RNA-seq libraries were sequenced on the HiSeq-2500 platform at Novogene.

### RNA-seq data processing

The sequencing reads were processed for quality trimming using the Trim Galore (version 0.6.7), and subsequently aligned to the mouse mm9 reference genome using HISAT2 (version 2.2.1) [35]. Gene expression was calculated by StringTie (version 2.2.1) according to the refFlat database [36].

### ChIP-seq data processing

Raw sequencing data were processed using Trim Galore (version 0.6.7) to remove adapter sequences and low-quality reads. The resulting high-quality paired-end reads were aligned to the mouse mm9, or human hg19 reference genome using Bowtie2 (version 2.4.5) with the following parameters: -t -q -N 1 -L 25 [37]. PCR duplicates and multiple-mapped reads were removed using JavaScript (MarkDuplicates.jar). Uniquely mapped reads were converted to BigWig format for visualization on the UCSC Genome Browser using deepTools (version 3.5.1) [38]. After validating the reproducibility, biological replicates were pooled for downstream analysis.

### ChIP-seq data normalization

To ensure accurate cross-sample comparisons of ChIP-seq signals across different batches, we normalized Pol II occupancy using a background-corrected RPKM approach. Specifically, we calculated the average RPKM signal of Pol II within a 1-kb window centered on the transcription start site (TSS). As a background reference, we measured the signal within the upstream region from –4 to –3 kb relative to the TSS. To mitigate gene-specific background effects, the mean background signal across all genes was used to derive a background coefficient. Normalized Pol II RPKM values were obtained by dividing the promoter RPKM by this coefficient. The same strategy was applied to Ser2p signals, with normalization performed over the gene body region.

### Identification of germ layer-specific genes

We utilized the normalized RPKM of Ser2p to identify lineage-specific expressed genes in germ layers. A gene was classified as germ-layer-specific if its normalized Ser2p RPKM > 3 in one germ layer and remained < 2 in all others. To account for developmental similarity among layers, we allowed expression above three in one additional layer. A similar approach was used for RNA-seq data, defining lineage-specific genes as those with expression levels > 5 in one germ layer and < 5 in the others. The complete list of germ layer–specific genes is provided in Supplementary Table Sheet 3.

### Identification of transcriptionally decoupled genes

Transcriptionally decoupled genes were identified using the following criteria: normalized RPKM for Ser2p within the gene body > 2, and FPKM < 1 as measured by RNA-seq. Genes with another annotated gene located within 10 kb of their genomic region were excluded to avoid potential overlap or ambiguous assignment. All identified decoupled genes are listed in Supplementary Table Sheet 4.

### Identification of bivalent genes and pol II (+) bivalent genes

Bivalent genes were identified based on public ChIP-seq data (GSE125318). Genes were defined as bivalent if their promoters exhibited normalized RPKM values > 2 for both H3K4me3 and H3K27me3. The resulting gene list is provided in Supplementary Table Sheet 5. Bivalent genes with normalized Pol II RPKM > 3 were classified as Pol II (+) bivalent genes (Supplementary Table Sheet 6). Among the remaining genes, promoter-associated Pol II normalized RPKM > 3 was used as the threshold to define activated genes, whereas genes below this cutoff were classified as silenced.

### Clustering analysis

K-means clustering of gene expression, histone modifications, Pol II, and Ser2p signals was performed using Cluster 3.0 with uncentered correlation as the distance metric. Hierarchical clustering was conducted based on Spearman’s rank correlation. Clustering results were visualized using Java TreeView.

### GO analysis

GO term enrichment was conducted using the DAVID functional annotation tool (https://davidbioinformatics.nih.gov/) with the standard mouse genome as background. GO terms with a P-value < 0.01 were considered statistically significant and reported in the corresponding figures.

### Statistical analyses

Statistical significance was assessed using two-tailed unpaired Student’s t-tests unless otherwise specified. Box plots display medians, interquartile ranges (boxes), and 1.5 × interquartile range whiskers. Statistical analyses were performed using R (v4.3.1). Detailed statistical tests and sample sizes are indicated in the figure legends.

## Results

### Genome-wide profiling of RNA Pol II in mouse early post-implantation embryos

To investigate the dynamics of RNA Pol II in mouse post-implantation embryos, we first tested the sensitivity of antibodies to capture total RNA Pol II (referred to hereafter as Pol II) at low-input cell level using STAR ChIP-seq [13]. The distribution of Pol II in 100k and 10k HeLa cells was highly comparable to that of the ENCODE data (Supplementary Fig. S1A and B) [39]. Around 79% and 68% Pol II peaks in reference data were recaptured by STAR ChIP-seq, respectively (Supplementary Fig. S1C). We then separated mouse E6.5 embryos into epiblast (Epi), visceral endoderm (VE), extra-embryonic ectoderm (ExE), and E7.5 embryos into ectoderm (Ect), mesoderm (Mes), endoderm (End), and ExE according to the method we previously reported [32]. ChIP-seq data for Pol II and its Ser2-phosphorylated form (hereafter as Ser2p) were generated. To validate the reliability of our data, we also performed RNA-seq for the corresponding stages. We confirmed that these data are highly reproducible (Supplementary Fig. S1D). Hierarchical clustering analysis revealed that Pol II and Ser2p signals are similar between replicates but are distinct among different lineages (Supplementary Fig. S1E). We then pooled data from replicates in the subsequent analyses.

**Figure 1.**
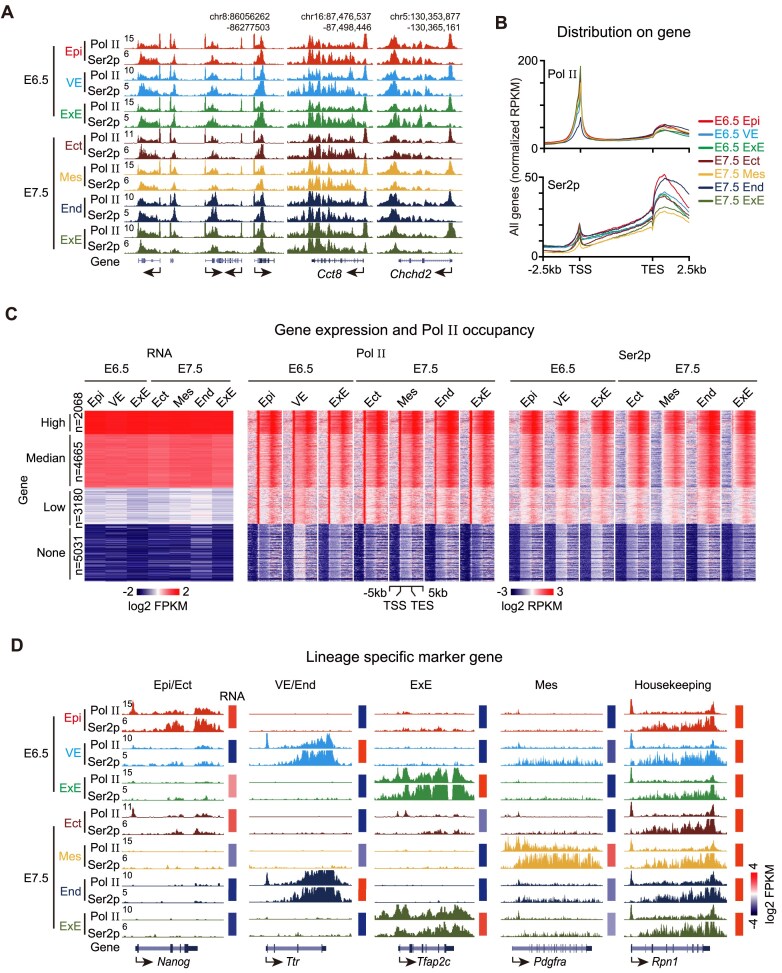
Genome-wide landscape of RNA Pol II and Ser2p in early post-implantation mouse embryos. (**A**). UCSC genome browser snapshots showing Pol II and Ser2p occupancy across representative genomic loci in distinct germ layers at E6.5 and E7.5. Genomic coordinates are indicated above each track. (**B**). Average plots illustrating the distribution of Pol II (top) and Ser2p (bottom) at gene bodies and their flanking regions. (**C**). Heatmaps of RNA expression (left), Pol II (middle), and Ser2p (right) signals at gene bodies across germ layers. Genes are stratified into four groups based on RNA expression levels across all germ layers using k-means clustering. (**D**). Representative lineage-specific marker genes showing Pol II and Ser2p enrichment by genome browser view. Corresponding gene expression levels are shown as heatmaps (right). Housekeeping gene *Rpn1* is included as a control.

Generally, Pol II was highly enriched at transcription start sites (TSS) and transcribed gene bodies, whereas Ser2p showed weaker TSS distribution but stronger presence at gene bodies and 3′ transcription end site (TES) regions at the genome-wide level, consistent with previous reports (Fig. 1A and B) [40]. The number of Pol II-positive and Ser2p-positive genes was comparable across embryonic tissues (Supplementary Fig. S1F). We categorized gene expression levels into high, medium, low, and non-expressed groups across embryonic tissues by RNA-seq. The result showed a strong positive correlation between the enrichment of Pol II and Ser2p and gene expression levels (Fig. 1C). Analysis of lineage-specific marker genes strongly supported the correct identities of mouse embryonic tissues and the dynamic Pol II and Ser2p landscapes in each germ layer (Fig. 1D). Taken together, these data demonstrated the high quality of Pol II and Ser2p data we generated in early post-implantation embryos.

### Transcriptionally decoupled genes during gastrulation

**Figure 2.**
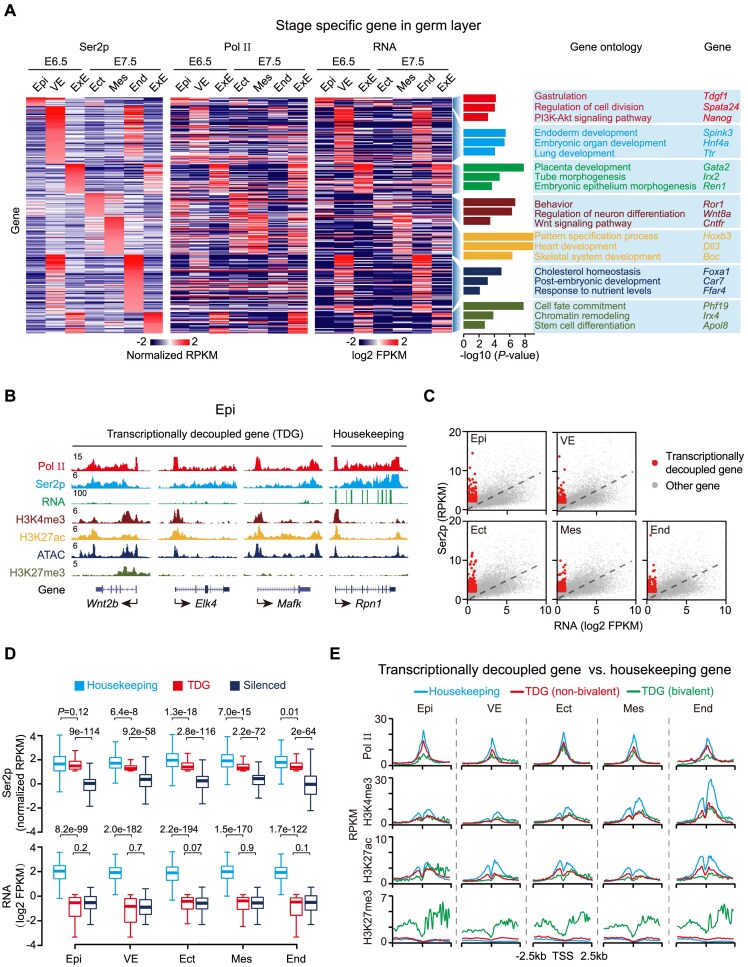
Transcriptionally decoupled genes exhibit Ser2p enrichment despite low mRNA output. (**A**). Heatmaps showing lineage-specific genes identified by Ser2p enrichment across germ layers at E6.5 and E7.5. Corresponding Pol II promoter occupancy and RNA expression are shown for the same gene sets. Gene Ontology (GO) terms are annotated on the right. (**B**). Genome browser snapshots depicting chromatin features of representative transcriptionally decoupled genes, characterized by high Ser2p signal but low RNA expression. Housekeeping gene *Rpn1* is shown as a control. (**C**). Scatter plots showing the correlation between Ser2p and RNA levels of all genes in E6.5 and E7.5 embryos. (**D**). Box plots comparing Ser2p signal (top) and RNA expression (bottom) between transcriptionally decoupled, housekeeping, and silenced genes. Center lines represent medians; box limits indicate the 25th and 75th percentiles; whiskers extend to 1.5 times the interquartile range (IQR). *P*-values were calculated using an unpaired two-tailed Student’s *t*-test. (**E**). Line plots showing the distribution of Pol II, H3K4me3, H3K27ac, and H3K27me3 at promoters of transcriptionally decoupled versus housekeeping genes. Transcriptionally decoupled genes are further subclassified into bivalent and non-bivalent groups.

To explore the relationship between transcriptional activity and cell fate specification, we analyzed RNA-seq and ChIP-seq data to identify lineage-specifically expressed genes and Pol II dynamics across mouse germ layers. We found that both Pol II occupancy and Ser2p levels correlated strongly with gene expression (Supplementary Fig. S2A). Genome-wide profiling further revealed that Ser2p distribution exhibits clear lineage specificity at both E6.5 and E7.5 (Fig. 2A). Genes with stage- and lineage-specific Ser2p enrichment were enriched for functions related to cell fate determination. For instance, in the E6.5 epiblast, Ser2p is preferentially associated with genes involved in gastrulation and PI3K-Akt signaling pathways, whereas in the visceral endoderm, Ser2p-marked genes are primarily linked to endoderm and lung development (Fig. 2A).

Unexpectedly, a subset of genes exhibited robust Ser2p enrichment despite having minimal detectable mRNA expression, as measured by Smart-seq2 (Fig. 2B and C). These transcriptionally decoupled genes ranged from 155 to 375 across different germ layers (Supplementary Fig. S2B). A global analysis showed that their Ser2p signal intensities are comparable to those of housekeeping genes but have markedly lower expression levels (Fig. 2D). This pattern is reproducible in both a published mRNA-seq dataset and our own total RNA-seq from E6.5 epiblast (Supplementary Fig. S2C), ruling out artifacts due to polyadenylation bias [32].

Transcriptionally decoupled genes remained silent or lowly expressed until E9.5, but were subsequently upregulated in E14.5 and adult tissues (Supplementary Fig. S2D). Gene ontology analysis further revealed enrichment for future lineage-specific processes (Supplementary Fig. S2E). For instance, genes identified in E6.5 epiblasts are enriched for mesoderm development, while those in the ectoderm are related to forebrain development, suggesting that transcriptionally decoupled genes represent key regulators of lineage specification. Consistently, 12–24% of these genes are bivalent genes, a defining feature of important developmental regulators (Supplementary Fig. S2F). Chromatin profiling showed that most transcriptionally decoupled genes exhibited chromatin features largely comparable to housekeeping genes. In contrast, the bivalent subset of these genes displayed substantially elevated H3K27me3, accompanied by reductions in Pol II, H3K4me3, and H3K27ac at their promoters (Fig. 2E and Supplementary Fig. S2G). Notably, this chromatin configuration diverges from the previously described “primed state,” which is characterized by promoter-associated Pol II but lacks productive elongation. These results suggest a distinct chromatin environment associated with bivalent genes during cell fate specification in gastrulation.

### Dynamic recruitment of pol II at bivalent promoters

**Figure 3.**
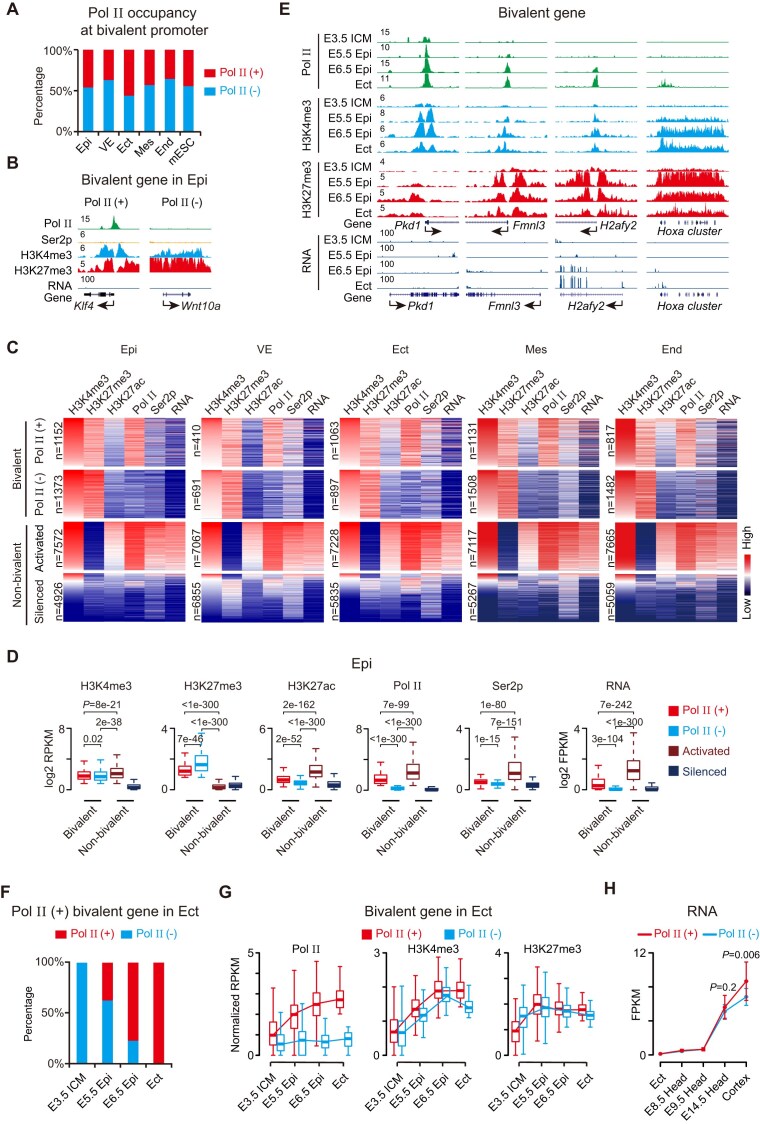
Stepwise recruitment of Pol II at bivalent promoters during lineage specification. (**A**). Bar plots showing the proportion of bivalent genes with or without Pol II occupancy across germ layers (GSE125318) and in mESCs (GSE31039). (**B**). Genome browser views of Pol II, Ser2p, H3K4me3, and H3K27me3 enrichment at representative Pol II (+) and Pol II (–) bivalent promoters. (**C**). Heatmaps showing H3K4me3, H3K27me3, H3K27ac, Pol II, Ser2p, and RNA levels in E6.5 and E7.5 embryos. Genes are grouped into four categories: Pol II (+), Pol II (–), activated, and silenced. (**D**). Boxplots showing H3K4me3, H3K27me3, H3K27ac, Pol II, Ser2p, and RNA levels in Epi for the same gene sets as in (**C**). (**E**). Representative genomic loci displaying progressive enrichment of H3K27me3, H3K4me3, and Pol II from ICM to Ect (GSE71434, GSE76687). (**F**). Bar plots quantifying Pol II (+) bivalent genes in E7.5 ectoderms and their Pol II binding status in earlier stages (ICM, E5.5 Epi, E6.5 Epi). (**G**). Box plots showing enrichment of Pol II, H3K4me3, and H3K27me3 at Pol II (+) and Pol II (–) bivalent promoters during development, corresponding to genes in panel F. (**H**). Line plots showing RNA expression dynamics of Pol II (+) versus Pol II (–) bivalent genes across developmental stages (E7.5 to 8-week adult cortex).

To investigate the timing of Pol II engagement at developmental promoters during gastrulation, we analyzed its distribution across distinct germ layers. Using a published dataset, we identified bivalent genes in Epi, VE, Ect, Mes, and End according to the enrichment of H3K4me3 and H3K27me3 (Supplementary Fig. S3A and B, Materials and methods), and examined Pol II distribution across these germ layers. Approximately 45% of bivalent genes exhibited Pol II enrichment at their promoters in germ layers and mouse embryonic stem cells (mESCs) (Fig. 3A). For simplicity, we classified bivalent promoters as Pol II (+) or Pol II (-) based on the presence or absence of Pol II binding (Fig. 3B). Although Pol II occupancy at Pol II (+) bivalent promoters was clearly higher than at Pol II (−) bivalent promoters, it remained lower than that at fully activated promoters (Fig. 3C and D). Both Pol II (+) and Pol II (−) bivalent promoters exhibited similar chromatin features across developmental stages, with comparable H3K4me3 levels, slightly lower H3K27me3 at Pol II (+) promoters, and minimal H3K27ac, Ser2p, and RNA expression, consistent with a lack of productive transcription (Fig. 3C and D).

Pol II (+) promoters are dynamically remodeled across developmental transitions, with over 30% being newly established during germ layer specification (Supplementary Fig. S3C). For instance, from Epi to Ect, 394 Pol II (+) promoters are newly established, while 483 promoters that were previously Pol II (+) no longer retain (Supplementary Fig. S3D). The gained Pol II (+) promoters are associated with the next stage of lineage specification, while lost Pol II (+) promoters are involved in lineage-inappropriate functions (Supplementary Fig. S3E). Interestingly, Pol II (+) promoters are progressively established during the transition from the epiblast to germ layer specification. H3K27me3 is first deposited by E5.5, followed by a gradual increase in H3K4me3 at E6.5 and the subsequent recruitment of Pol II (Fig. 3E–G and Supplementary Fig. S3F). Notably, Pol II (+) genes tend to be more robustly activated than their Pol II (−) counterparts (Fig. 3H and Supplementary Fig. S3G), highlighting the functional relevance of Pol II recruitment during lineage commitment.

### KMT2B/H3K4me3 and EED/H3K27me3 regulate Pol II recruitment

**Figure 4.**
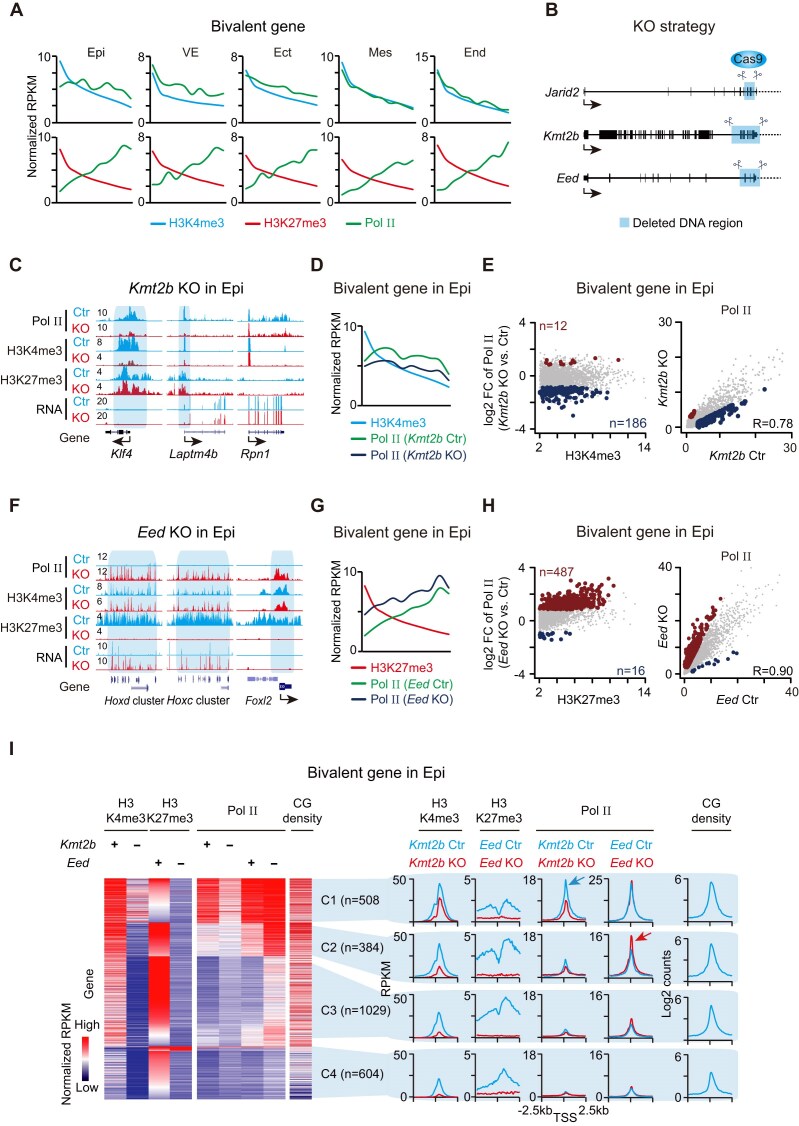
Pol II recruitment at bivalent promoters is regulated by H3K4me3 and H3K27me3. (**A**). Average plots ranking bivalent promoters in Epi, VE, Ect, Mes, and End by H3K4me3 (top) or H3K27me3 (bottom) signal, with corresponding Pol II occupancy patterns. (**B**). A schematic diagram illustrates the knockout strategy for *Jarid2, Kmt2b*, and *Eed* using CRISPR-Cas9. Target exons are highlighted by shadow boxes. (**C**). Genome browser tracks showing the enrichment of Pol II, H3K4me3, and H3K27me3 in control and *Kmt2b* knockout Epi. Highlighted regions denote decreased Pol II occupancy. Housekeeping gene *Rpn1* serves as a control. (**D**). Average plots ranking bivalent promoters in E6.5 epiblasts by H3K4me3 signal. Pol II signals from control and *Kmt2b* knockout embryos are correspondingly shown. (**E**). Left: Scatter plot showing correlation between promoter H3K4me3 and fold change of Pol II enrichment in epiblast (*Kmt2b* knockout vs. control). Right: Scatter plot comparing Pol II signals between control and *Kmt2b* knockout embryos in epiblast. (**F**). Genome browser tracks showing Pol II, H3K4me3, and H3K27me3 in *Eed* knockout and control epiblasts. Highlighted regions denote increased Pol II occupancy. (**G**). Average plots ranking bivalent promoters in E6.5 epiblasts by H3K27me3 signal. Pol II signals from control and *Eed* knockout embryos are correspondingly shown. (**H**). Left: Scatter plot showing correlation between promoter H3K27me3 and fold change of Pol II enrichment in epiblast (*Eed* knockout vs. control). Right: Scatter plot comparing Pol II signals between control and *Eed* knockout embryos in the epiblast. (**I**). Heatmaps and line plots showing H3K4me3, H3K27me3, Pol II, and CpG density across four clusters (C1–C4) of bivalent promoters defined by chromatin signature. Changes in Pol II occupancy in response to *Kmt2b* or *Eed* knockout are shown for each cluster. Arrows indicate up-regulation (lower–right) or down-regulation (upper–left).

To dissect the chromatin features associated with Pol II recruitment, we ranked bivalent genes by the promoter enrichment of H3K4me3 and H3K27me3, and examined Pol II enrichment across these ranked groups. Pol II occupancy was positively correlated with H3K4me3 levels and negatively correlated with H3K27me3 (Fig. 4A). Specifically, bivalent promoters with high H3K4me3 and relatively low H3K27me3 were preferentially bound by Pol II, as exemplified by genes such as *H2afy2* and *Hoxa* cluster in Ect, where Pol II engagement was markedly stronger compared to E6.5 epiblasts. (Fig. 3E).

To functionally test the role of histone modifiers in Pol II occupancy, we generated *Jarid2, Kmt2b*, and *Eed* knockout embryos by CRISPR-Cas9-mediated gene editing of zygotes [33] (Fig. 4B). RNA-seq confirmed successful deletion of the targeted exons (Supplementary Fig. S4A). As expected, *Kmt2b* knockout resulted in a global reduction of H3K4me3 at bivalent genes, while *Eed* deficiency abolished H3K27me3 deposition (Supplementary Fig. S4B). Conversely, H3K27me3 and H3K4me3 levels remained largely unchanged in *Kmt2b* and *Eed* knockout embryos, respectively. We found *Jarid2* is dispensable for Pol II recruitment at bivalent loci in both Epi and ExE, differing from its reported function in embryonic stem cells (Supplementary Fig. S4C–H). In contrast, *Kmt2b* deficiency led to a modest decrease in Pol II occupancy at a subset of bivalent genes in both Epi (Fig. 4C–E) and in ExE (Supplementary Fig. S4F and G), while RNA expression of these genes remained largely unchanged (Fig. 4C and Supplementary Fig. S5A). Importantly, *Eed* knockout led to a significant enrichment of Pol II at bivalent gene promoters in both Epi (Fig. 4F–H) and ExE (Supplementary Fig. S4F–H), resulting in subsequent gene activation at the *Hox* cluster and other developmental genes (Fig. 4F and Supplementary Fig. S5A), with Pol II occupancy and RNA levels remaining lower than those at fully activated genes. These findings support a model in which H3K4me3 promotes, and H3K27me3 antagonizes, Pol II recruitment during early embryogenesis.

To further delineate the chromatin logic underlying Pol II recruitment, we stratified bivalent promoters into four categories based on H3K4me3 and H3K27me3 enrichment: strong H3K4me3 and weak H3K27me3 (cluster 1, C1), strong H3K4me3 and strong H3K27me3 (C2), weak H3K4me3 and strong H3K27me3 (C3), and weak H3K4me3 and weak H3K27me3 (C4). In control embryos, we observed significant enrichment of Pol II at C1 promoters (Fig. 4I and Supplementary Fig. S5B). Genetic ablation of *Kmt2b* led to a marked reduction in Pol II occupancy at C1 promoters, while *Eed* deficiency conversely caused substantial Pol II accumulation at C2 and a weaker increase at C3 promoters (Fig. 4I and Supplementary Fig. S5C). Although CpG islands are associated with the deposition of both H3K4me3 and H3K27me3 [41], our analysis indicates that CG density alone does not account for differences in Pol II occupancy. Instead, Pol II recruitment appeared to be determined by the relative strengths of H3K4me3 and H3K27me3 at promoters (Fig. 4I and Supplementary Fig. S5B). *Kmt2b* knockout had minimal effects on RNA expression, consistent with the transcriptionally repressed nature of bivalent genes, whereas *Eed* deficiency resulted in increased transcription of genes in the C2 and C3 clusters, accompanied by elevated Pol II occupancy (Supplementary Fig. S5D). Together, these findings identify KMT2B and EED as opposing chromatin regulators that modulate Pol II recruitment at bivalent promoters through differential deposition of H3K4me3 and H3K27me3.

### SP1 facilitates Pol II deposition in three germ layers

**Figure 5.**
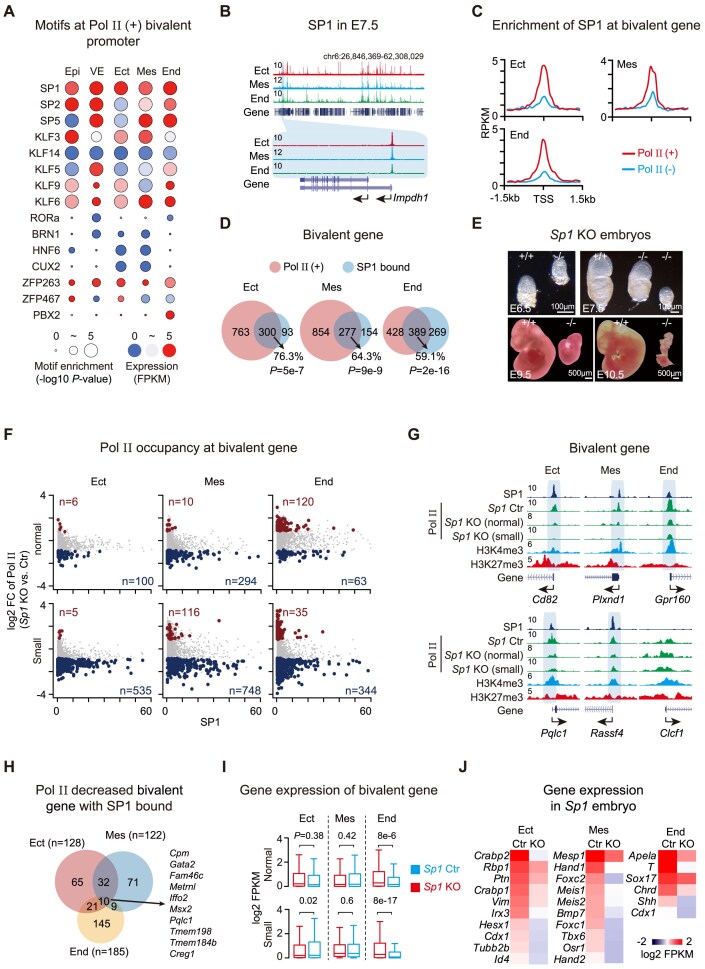
SP1 promotes Pol II recruitment at bivalent promoters in germ layers. (**A**). Transcription factor motif enrichment at Pol II (+) bivalent promoters in each germ layer. Circle size reflects motif enrichment; color indicates TF expression levels. (**B**). Genome browser tracks showing SP1 occupancy at representative loci across ectoderm, mesoderm, and endoderm. A zoomed view of *Impdh1* is shown. (**C**). Line plots show the enrichment of SP1 at Pol II (+) and Pol II (–) bivalent genes in three germ layers. (**D**). Venn diagrams showing overlap between SP1-bound and Pol II (+) bivalent genes across ectoderm, mesoderm, and endoderm. (**E**). Brightfield images of *Sp1* control and knockout embryos from E6.5 to E10.5, with knockout embryos at E7.5 displaying either normal (*n* = 6) or small size (*n* = 20). (**F**). Scatter plot showing the correlation between promoter SP1 binding and Pol II enrichment fold change at E7.5 bivalent promoters (*Sp1* knockout vs. control). (**G**). Genome browser tracks showing SP1, H3K4me3, H3K27me3, and Pol II signals at representative loci in control and *Sp1* knockout embryos. Pol II-depleted regions upon *Sp1* loss are highlighted. *Clcf1* is shown as a negative control lacking SP1 binding. (**H**). Venn diagram showing the overlap of SP1-bound bivalent genes exhibiting reduced Pol II occupancy across germ layers after *Sp1* deletion. (**I**). Box plots comparing RNA expression levels of bivalent genes between *Sp1* control and knockout embryos at E7.5. (**J**). Heatmaps showing differential gene expression in *Sp1* knockout small embryos relative to controls.

To identify transcription factors that may mediate Pol II recruitment at bivalent promoters during early embryogenesis, we performed motif analysis using HOMER and found that Pol II (+) promoters are significantly enriched for distinct transcription factor motifs across germ layers (Fig. 5A) [42]. Notably, SP1 emerged as the most consistently enriched transcription factor in all tissues. We then examined the genomic distribution of SP1 in the three germ layers at E7.5 by STAR ChIP-seq (Fig. 5B). SP1 binding peaks were predominantly localized at promoter regions and exhibited strong enrichment for canonical SP1 motifs (Fig. 5B and Supplementary Fig. S6A and B), with approximately 10% of SP1 peaks located at bivalent gene promoters and nearly 80% at activated gene promoters (Supplementary Fig. S6C). We found that SP1 is more abundantly distributed on Pol II (+) promoters compared to that of Pol II (–) promoters (Fig. 5C). Consistently, bivalent genes bound by SP1 exhibited higher Pol II occupancy than those without SP1 binding, although still lower than that observed at activated promoters (Supplementary Fig. S6D). Most of bivalent promoters that were bound by SP1 were Pol II (+) promoters in Ect (76.3%, 300 out of 393), Mes (64.3%, 277 out of 431), and End (59.1%, 389 out of 658) (Fig. 5D). The limited overlap of SP1 target genes among germ layers suggests that SP1 regulates distinct transcriptional programs in a lineage-specific manner (Supplementary Fig. S6E).

We then constitutively deleted *Sp1* using the CRISPR-Cas9 knockout strategy as described above (Supplementary Fig. S6F and G). *Sp1* knockout embryos exhibited progressive developmental retardation with severe morphological defects and failed to survive beyond E10.5 (Fig. 5E and Supplementary Fig. S6H). The majority of *Sp1*-deficient embryos displayed decreased cell number and smaller size (small embryo, *n* = 20), while others developed normally (normal embryo, *n* = 6) at E7.5 (Fig. 5E). In *Sp1* knockout normal embryos, Pol II recruitment was modestly reduced in ectoderm and mesoderm. In contrast, small embryos displayed a marked reduction of Pol II occupancy at SP1-bound Pol II (+) promoters across all three germ layers (Fig. 5F and G). Specifically, 128, 122, and 185 genes showed reduced Pol II binding in ectoderm, mesoderm, and endoderm, respectively (Fig. 5H). Despite this, only 10 genes were shared across all layers, reinforcing the notion that SP1 regulates lineage-specific targets during germ layer specification (Fig. 5H).

RNA expression analysis showed that *Sp1* loss led to reduced transcription of a subset of genes. Among the downregulated genes in each germ layer, approximately 16–25% were bivalent genes (Supplementary Fig. S6I). Overall, transcriptional changes at bivalent genes were modest in ectoderm and mesoderm, but more pronounced in endoderm (Fig. 5I). Only a small proportion of downregulated genes (1.9–9.1%) were both SP1-bound and bivalent (Supplementary Fig. S6I). Notably, the downregulated genes in *Sp1* knockout germ layers included key developmental regulators, such as *Hesx1* and *Tubb2b* in ectoderm, *Mesp1* and *Hand1* in mesoderm, and *Sox17* in endoderm [43, 44] (Fig. 5J), indicating that SP1-mediated Pol II recruitment is critical for the activation of lineage-defining developmental genes.

Together, these results identify SP1 as a key transcription factor that facilitates Pol II recruitment to bivalent promoters in a tissue-specific manner. Loss of SP1 disrupts this process, impairs germ layer gene expression, and leads to developmental defects.

### SP1 facilitates Pol II occupancy in conjunction with reduced H3K27me3

**Figure 6.**
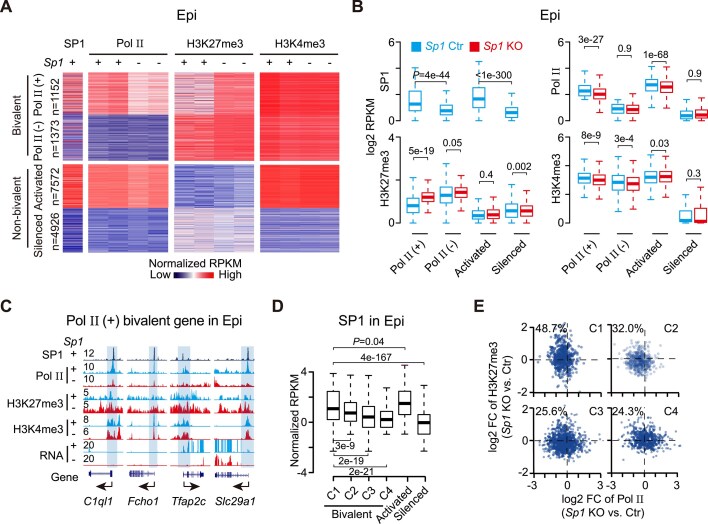
SP1 facilitates Pol II occupancy by antagonizing H3K27me3. (**A**). Heatmaps showing SP1, Pol II, H3K4me3, and H3K27me3 signals at Pol II (+), Pol II (–), activated and silenced promoters in control and *Sp1* knockout E6.5 epiblasts. (**B**). Box plots quantifying signal intensities of SP1, Pol II, H3K4me3, and H3K27me3 at Pol II (+), Pol II (–), activated and silenced promoters in *Sp1* knockout versus control epiblast. (**C**). Genome browser tracks displaying chromatin and gene expression changes at representative loci upon *Sp1* deletion. Increased H3K27me3 is highlighted. (**D**). Box plots showing SP1 binding across bivalent (C1–C4), activated, and silenced promoter clusters defined by chromatin states. (**E**). Dual-axis scatter plots showing fold changes of Pol II and H3K27me3 upon *Sp1* knockout in each cluster. The percentage of genes with reduced Pol II and increased H3K27me3 (upper-left quadrant) is indicated.

To elucidate the chromatin basis of SP1-mediated Pol II recruitment, we examined SP1 binding, Pol II occupancy, and histone modifications at bivalent gene promoters in the E6.5 epiblast. SP1 is preferentially enriched at Pol II (+) bivalent promoters, which also display relatively lower H3K27me3 levels and elevated H3K4me3 compared to Pol II (–) promoters (Fig. 6A). Upon *Sp1* knockout, Pol II levels were markedly reduced at Pol II (+) bivalent genes, while no obvious change was observed at Pol II (–) genes (Fig. 6A and B), whereas Ser2p levels at Pol II (+) bivalent promoters remained largely unchanged (Supplementary Fig. S7A and B). This reduction in Pol II was accompanied by a significant increase in H3K27me3 and a modest decrease in H3K4me3 at Pol II (+) promoters, suggesting a shift toward a repressive chromatin state (Fig. 6A–C).

SP1 binding was most enriched in C1, characterized by high H3K4me3 and low H3K27me3, and gradually declined across clusters with more repressive chromatin features (Fig. 6D). Cluster-specific changes in chromatin state were observed between the control and *Sp1*-deficient epiblast. 48.7% of C1 genes exhibited concomitant reductions in Pol II and increases in H3K27me3, consistent with a shift toward a repressive state (Fig. 6E). In contrast, clusters C3 and C4, which bear high/low H3K27me3 and low H3K4me3, showed limited sensitivity to *Sp1* depletion.

Although RNA levels at bivalent genes were generally unchanged in *Sp1* knockout epiblast at E6.5, a subset of SP1-bound bivalent genes was preferentially downregulated upon *Sp1* knockout (Supplementary Fig. S7C, left). A similar trend was observed for Pol II (+) bivalent genes, in which SP1-bound targets exhibited decreases in Pol II and transcription (Supplementary Fig. S7D). At representative loci such as *Tfap2c* and *Slc29a1*, loss of *Sp1* resulted in coordinated reductions of Pol II occupancy and mRNA level (Fig. 6C). Gene ontology analysis of downregulated bivalent targets highlighted essential developmental pathways, including IGF1R and PI3K signaling as well as developmental growth (Supplementary Fig. S7C). Together, these results suggested that SP1 is associated with enhanced Pol II occupancy at bivalent promoters in conjunction with reduced H3K27me3, thereby contributing to the transcriptional regulation of developmental genes.

## Discussion

In this study, we delineate the chromatin-based regulatory mechanisms that orchestrate RNA polymerase II recruitment at developmental genes during early mouse embryogenesis. Our findings underscore the pivotal role of the dynamic interplay between histone modifications and transcription factors in establishing transcriptional competence at bivalent promoters. In particular, we demonstrate that the balance between activating inputs, such as H3K4me3 and the transcription factor SP1, and the repressive influence of H3K27me3 critically determines Pol II occupancy during lineage specification. This finely tuned antagonism highlights a fundamental epigenetic logic wherein transcriptional readiness is actively maintained while preventing untimely gene activation during sensitive windows of development.

Bivalent chromatin maintains developmental genes in a transcriptionally poised state, characterized by promoter-bound Pol II that lacks Ser2p and therefore does not support productive elongation or mRNA output [22, 45]. Our identification of transcriptionally decoupled genes uncovers a distinct subset of bivalent genes that exhibit ser2p signals across gene bodies without producing mature transcripts, extending transcriptional regulation beyond the canonical poised state in early embryonic development. These genes are enriched for key developmental regulators, suggesting functional relevance during lineage specification. Several mechanisms may underlie this uncoupling, including inefficient RNA processing, delayed transcript release, enhanced nuclear RNA degradation by the RNA exosome, or the production of short RNA species [46–51]. However, the precise molecular mechanisms and functional consequences of this atypical transcriptional state remain to be determined.

Temporal chromatin analyses further elucidate the sequential nature of bivalency establishment. H3K27me3 is deposited early at the E5.5 epiblast to ensure transcriptional repression of developmental regulators. Subsequently, H3K4me3 accumulation and transcription factor binding promote the formation of a bivalent chromatin state that is permissive for Pol II recruitment. PRC2-mediated repression contributes to safeguarding developmental gene expression by preventing inappropriate activation [52]. Notably, the removal of H3K27me3, as observed in *Eed*-deficient embryos, enhances Pol II occupancy but only at a subset of bivalent promoters. This observation suggests that de-repression alone is insufficient for Pol II engagement; additional activating cues are required to initiate transcriptional activation.

Our genetic perturbation studies reveal that knockout of *Kmt2b* does not completely abolish Pol II recruitment at bivalent promoters. This partial effect likely reflects functional compensation by SET1A, another H3K4 methyltransferase, which may sustain residual H3K4me3 levels and partially preserve Pol II recruitment [53, 54]. Importantly, SP1 emerges as a critical modulator of this process, facilitating Pol II loading by counteracting H3K27me3 deposition. The lineage-specific distribution of SP1-bound targets across the three germ layers suggests that SP1 operates in concert with tissue-specific cofactors or chromatin environments to direct germ-layer-specific gene expression programs in addition to its intrinsic DNA-binding specificity [55, 56]. Consistent with this cooperative model, Pol II occupancy decreases at only a subset of SP1-bound promoters upon *Sp1* loss, suggesting compensatory support from other transcriptional and chromatin regulators. The limited impact of *Kmt2b* and *Sp1* deficiency on the overall expression of bivalent genes is consistent with the inherently restrained transcriptional state of bivalent chromatin, in which promoter-bound Pol II does not engage in productive elongation. Nevertheless, the selective downregulation of key developmental regulators highlights a critical role for SP1 in conferring transcriptional competence at these loci, thereby contributing to the precise regulation of gene expression during embryonic cell fate specification.

While our study provides key insights into the regulatory axis linking chromatin state and Pol II dynamics during early embryogenesis, several limitations remain. First, SP1 binding is associated with reduced H3K27me3 and enhanced Pol II occupancy at bivalent promoters, the precise mechanistic link remains to be established. Second, our analyses are confined to discrete developmental time points, which may not fully capture the continuous and dynamic nature of transcriptional regulation throughout embryogenesis. Moreover, although embryos were separated into individual germ layers to substantially reduce cellular complexity, residual heterogeneity cannot be completely excluded. Future studies leveraging live imaging and single-cell multi-omics will be essential to resolve the temporal coordination of chromatin remodeling, Pol II recruitment, and transcriptional output at single gene resolution.

In sum, our findings reveal a chromatin-based framework in which the recruitment of Pol II to bivalent promoters is governed by the opposing actions of H3K4me3 and H3K27me3, modulated by lineage-specific transcription factors such as SP1. This model not only refines our understanding of transcriptional regulation during mammalian gastrulation but also provides a conceptual foundation for dissecting gene regulation in broader developmental and pathological contexts.

## Supplementary Material

gkag305_Supplemental_Files

## Data Availability

The ChIP-seq and RNA-seq datasets generated in this study have been deposited in the NCBI Gene Expression Omnibus (GEO) with accession numbers GSE269652 and GSE269651, respectively. The sequencing information is provided in Supplementary Table 7.
