# Supplementary material for: SP1 antagonizes H3K27me3 to shape chromatin landscapes for RNA polymerase II recruitment during gastrulation
Source: Nucleic Acids Res. 2026 Apr 13;54(7):gkag305. doi: 10.1093/nar/gkag305 (PMC13076215; doi:10.1093/nar/gkag305)
Supplement: gkag305_Supplemental_Files [file gkag305_supplemental_files.zip › Supplemental data.pdf]

Supporting information for

SP1 antagonizes H3K27me3 to shape chromatin landscapes for RNA polymerase II recruitment during gastrulation

Xipeng Shen[1,2,†], Yuting Wen[1,2,†], Xiaohan Tang[3,†], Yujiao Liu[2,†], Wensi Li[2], Wenhao Chen[2], Shuheng Yang[2], Lidan Wang[2], Haibo Yang[4], Kunyan Liu[2,*], Lei Li[5,6,*], Yunlong Xiang[1,2,3,*]

[1]Department of Rheumatology & Immunology Children's Hospital of Chongqing Medical University, National Clinical Research Center for Children and Adolescents' Health and Diseases, Ministry of Education Key Laboratory of Child Development and Disorders. Chongqing Key Laboratory of Child Rare Diseases in Infection and Immunity, School of Basic Medical Sciences, Chongqing 400010, China

[2]Center for Medical Epigenetics, School of Basic Medical Sciences, Chongqing Medical University, Chongqing 400010, China

[3]Department of Reproductive Medicine Center, The First Affiliated Hospital of Chongqing Medical University, Chongqing 400010, China

[4]Information Center of Chongqing Medical University, Chongqing 400010, China

[5]State Key Laboratory of Stem Cell and Reproductive Biology, Key Laboratory of Organ Regeneration and Reconstruction, UCAS/IOZ/CAS, Beijing 100101, China

[6]Beijing Institute of Stem Cell and Regenerative Medicine, Beijing 100100, China

[†]These authors contributed equally

[*]To whom correspondence should be addressed. Email: xiangyl@cqmu.edu.cn

Correspondence may also be addressed to Lei Li. Email: lil@ioz.ac.cn

Correspondence may also be addressed to Kunyan Liu. Email: liuky@cqmu.edu.cn

Figure S1

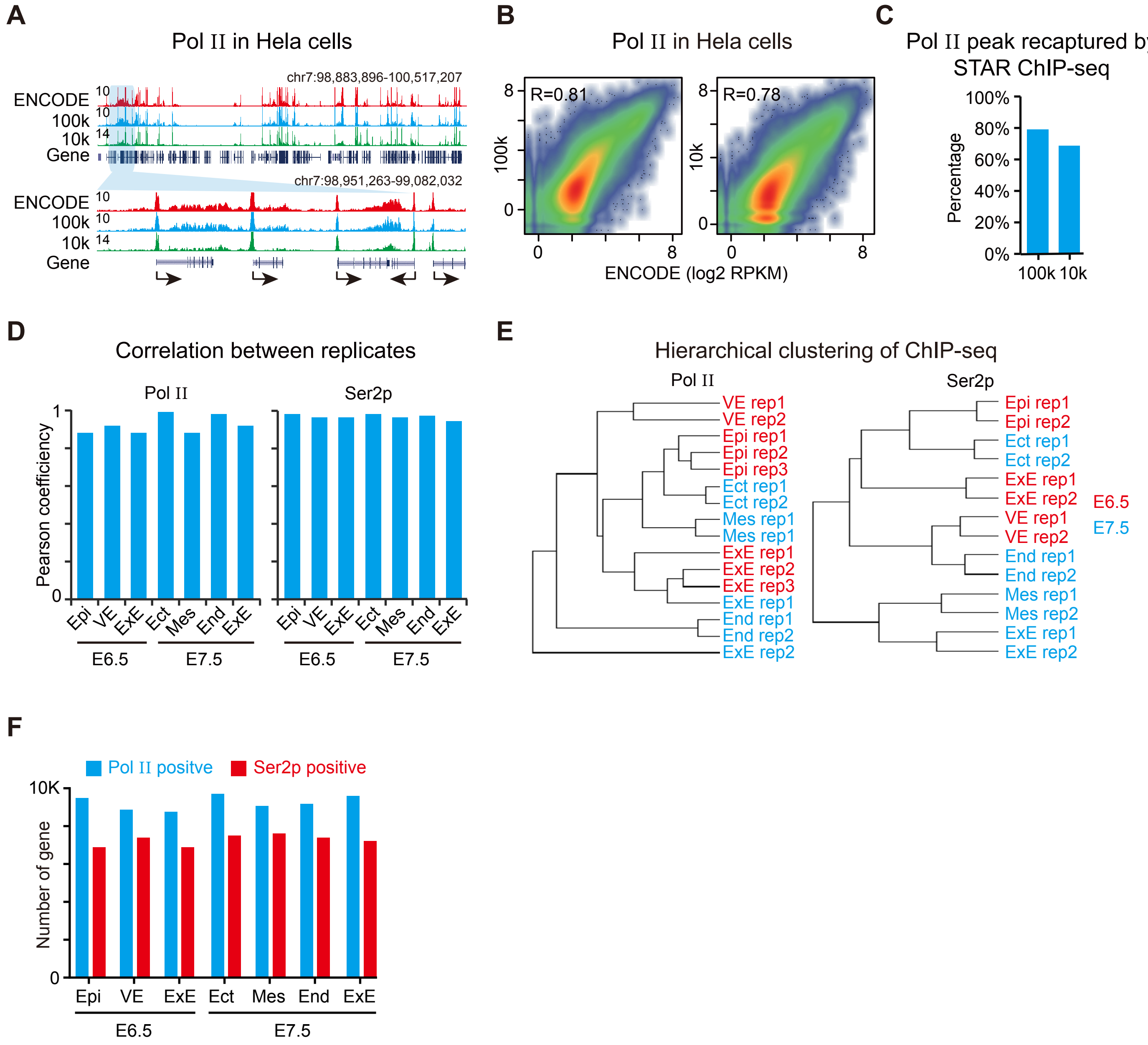

A
Pol II in Hela cells
chr7:98,883,896-100,517,207
ENCODE
100k
10k
Gene
chr7:98,951,263-99,082,032
B
Pol II in Hela cells
R=0.81
R=0.78
100k
10k
ENCODE (log2 RPKM)
C
Pol II peak recaptured by STAR ChIP-seq
Percentage
100k 10k
D
Correlation between replicates
Pol II
Ser2p
Pearson coefficiency
Epi VE ExE Ect Mes End ExE
E6.5
E7.5
E
Hierarchical clustering of ChIP-seq
Pol II
VE rep1
VE rep2
Epi rep1
Epi rep2
Epi rep3
Ect rep1
Ect rep2
Mes rep1
Mes rep1
ExE rep1
ExE rep2
ExE rep3
ExE rep1
End rep1
End rep2
ExE rep2
Ser2p
Epi rep1
Epi rep2
Ect rep1
Ect rep2
ExE rep1
ExE rep2
VE rep1
VE rep2
End rep1
End rep2
Mes rep1
Mes rep2
ExE rep1
ExE rep2
E6.5
E7.5
F
Pol II positve
Ser2p positive
10K
Number of gene
Epi VE ExE Ect Mes End ExE
E6.5
E7.5

Figure S1. Profiling of Pol II and Ser2p in mouse early lineages by STAR ChIP-seq. (**A**). Genome browser snapshots showing Pol II distribution in 100k and 10k HeLa cells using STAR ChIP-seq, with ENCODE (GSE31477) data shown as reference. (**B**). Scatter plots showing correlations between Pol II signals obtained by STAR ChIP-seq and ENCODE data. (**C**). Percentage of ENCODE Pol II peaks recaptured by STAR ChIP-seq in 100k and 10k HeLa cells. (**D**). Pearson correlation coefficients between biological replicates for Pol II (left) and Ser2p (right) ChIP-seq in E6.5 and E7.5 embryos. (**E**). Hierarchical clustering of Pol II and Ser2p ChIP-seq replicates across germ layers at E6.5 and E7.5. (**F**). Number of genes with detectable Pol II or Ser2p signal across germ layers at E6.5 and E7.5.

Figure S2

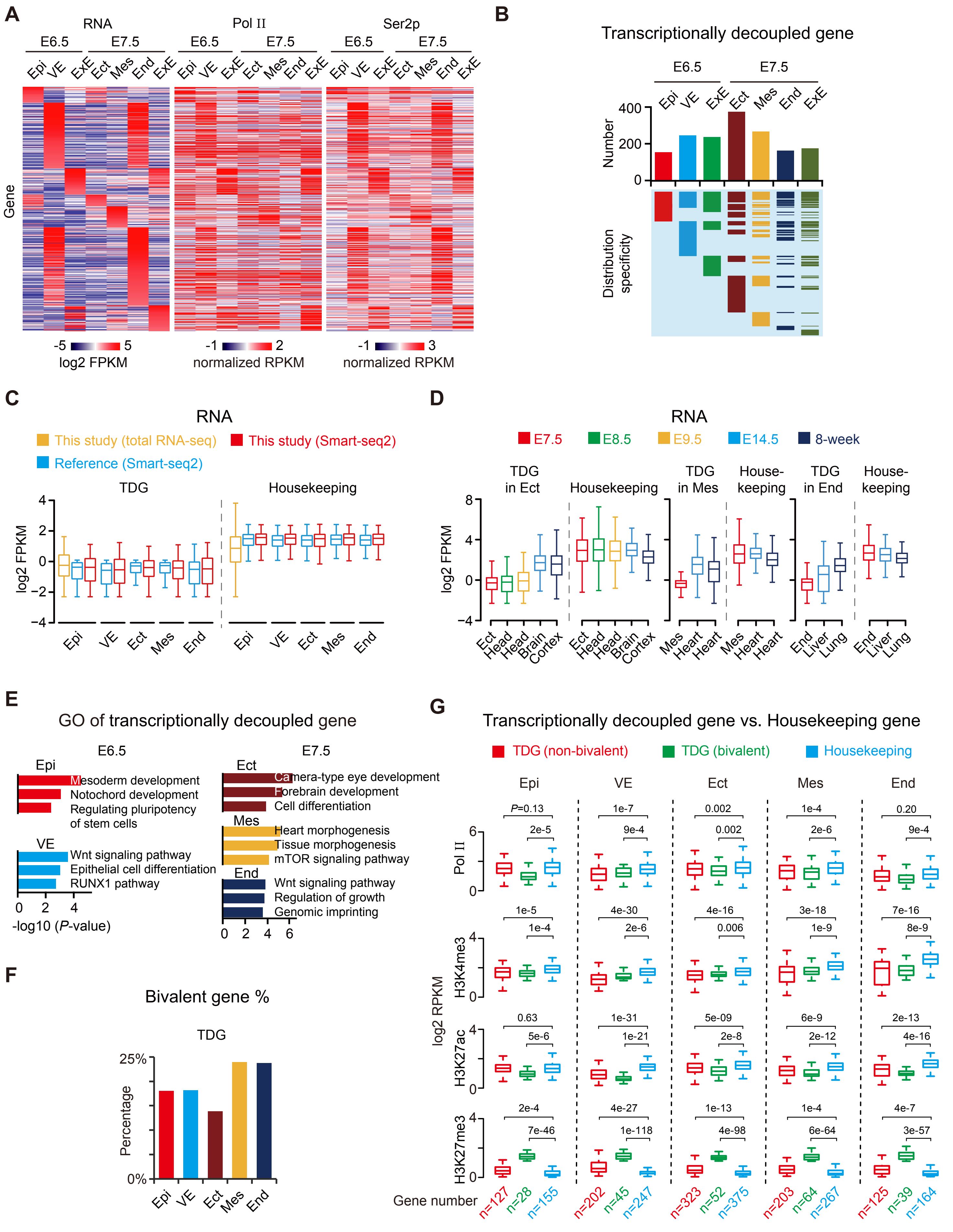
A
RNA
Pol II
Ser2p
E6.5
E7.5
Epi VE ExE Ect Mes End ExE
Gene
-5 5
log2 FPKM
-1 2
normalized RPKM
-1 3
normalized RPKM
B
Transcriptionally decoupled gene
E6.5
E7.5
Epi VE ExE Ect Mes End ExE
400
200
0
Number
Distribution specificity
C
RNA
This study (total RNA-seq)
This study (Smart-seq2)
Reference (Smart-seq2)
TDG
Housekeeping
log2 FPKM
4
2
0
−2
−4
Epi VE Ect Mes End Epi VE Ect Mes End
D
RNA
E7.5
E8.5
E9.5
E14.5
8-week
TDG in Ect
Housekeeping
TDG in Mes
House-keeping
TDG in End
House-keeping
8
4
0
−4
log2 FPKM
Ect Head Head Brain Cortex Ect Head Head Brain Cortex Mes Heart Heart Mes Heart Heart End Liver Lung End Liver Lung
E
GO of transcriptionally decoupled gene
E6.5
E7.5
Epi
Mesoderm development
Notochord development
Regulating pluripotency of stem cells
VE
Wnt signaling pathway
Epithelial cell differentiation
RUNX1 pathway
0 2 4
-log10 (P-value)
Ect
Camera-type eye development
Forebrain development
Cell differentiation
Mes
Heart morphogenesis
Tissue morphogenesis
mTOR signaling pathway
End
Wnt signaling pathway
Regulation of growth
Genomic imprinting
0 2 4 6
F
Bivalent gene %
TDG
25%
0%
Percentage
Epi VE Ect Mes End
G
Transcriptionally decoupled gene vs. Housekeeping gene
TDG (non-bivalent)
TDG (bivalent)
Housekeeping
Epi VE Ect Mes End
log2 RPKM
Pol II
6
0
P=0.13
2e-5
1e-7
9e-4
0.002
0.002
1e-4
2e-6
0.20
9e-4
H3K4me3
4
0
1e-5
1e-4
4e-30
2e-6
4e-16
0.006
3e-18
1e-9
7e-16
8e-9
H3K27ac
4
0
0.63
5e-6
1e-31
1e-21
5e-09
2e-8
6e-9
2e-12
2e-13
4e-16
H3K27me3
4
0
2e-4
7e-46
4e-27
1e-118
1e-13
4e-98
1e-4
6e-64
4e-7
3e-57
Gene number
n=127 n=28 n=155
n=202 n=45 n=247
n=323 n=52 n=375
n=203 n=64 n=267
n=125 n=39 n=164

Figure S2. Characteristics of transcriptionally decoupled genes during early development. (**A**). Heatmaps showing lineage specific genes identified by RNA-seq, with corresponding Pol II and Ser2p enrichment. (**B**). Bar plots showing the number and distribution of transcriptionally decoupled genes identified across different germ layers at E6.5 and E7.5. (**C**). Box plots comparing RNA expression of transcriptionally decoupled genes between this study and published E6.5 Epi data (GSE125318), with housekeeping genes as control. (**D**). Box plots showing RNA expression of transcriptionally decoupled genes across embryonic stages and tissues (GSE36026). (**E**). Gene ontology analysis showing biological processes associated with transcriptionally decoupled genes at E6.5 and E7.5. (**F**). Bar chart showing the proportion of bivalent genes among transcriptionally decoupled genes at E6.5 and E7.5. (**G**). Box plots showing the distribution of Pol II, H3K4me3, H3K27ac, and H3K27me3 at promoters of transcriptionally decoupled versus housekeeping genes. Transcriptionally decoupled genes are further subclassified into bivalent (green) and non-bivalent (red) groups. *P*-values were calculated using an unpaired two-tailed Student's t-test.

Figure S3

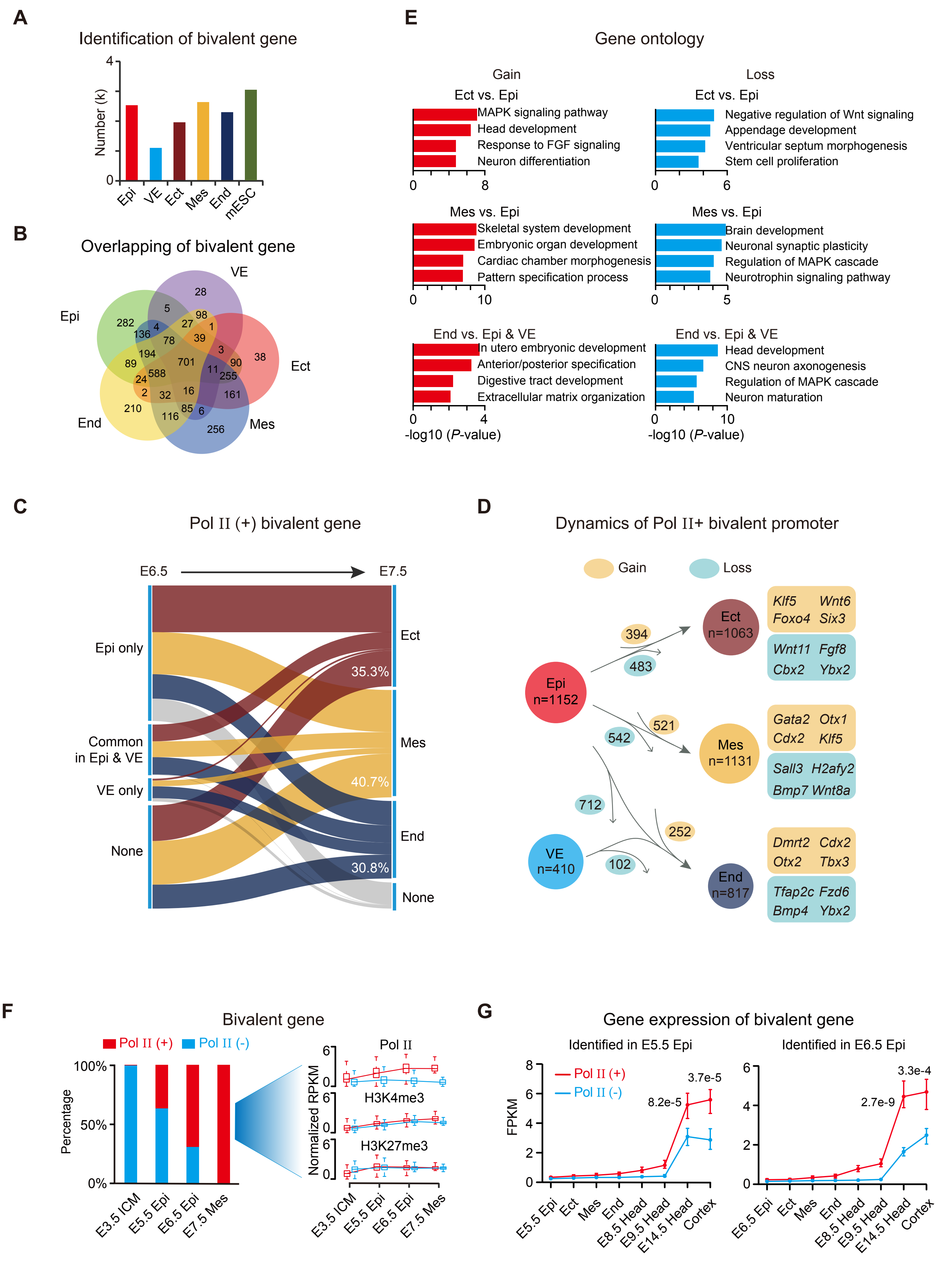
A
Identification of bivalent gene
Number (k)
Epi
VE
Ect
Mes
End
mESC
B
Overlapping of bivalent gene
Epi
VE
Ect
Mes
End
C
Pol II (+) bivalent gene
E6.5
E7.5
Epi only
Common in Epi & VE
VE only
None
Ect
Mes
End
None
35.3%
40.7%
30.8%
D
Dynamics of Pol II+ bivalent promoter
Gain
Loss
Epi n=1152
VE n=410
Ect n=1063
Mes n=1131
End n=817
394
483
521
542
712
252
102
Klf5 Wnt6 Foxo4 Six3
Wnt11 Fgf8 Cbx2 Ybx2
Gata2 Otx1 Cdx2 Klf5
Sall3 H2afy2 Bmp7 Wnt8a
Dmrt2 Cdx2 Otx2 Tbx3
Tfap2c Fzd6 Bmp4 Ybx2
E
Gene ontology
Gain
Loss
Ect vs. Epi
MAPK signaling pathway
Head development
Response to FGF signaling
Neuron differentiation
Negative regulation of Wnt signaling
Appendage development
Ventricular septum morphogenesis
Stem cell proliferation
Mes vs. Epi
Skeletal system development
Embryonic organ development
Cardiac chamber morphogenesis
Pattern specification process
Brain development
Neuronal synaptic plasticity
Regulation of MAPK cascade
Neurotrophin signaling pathway
End vs. Epi & VE
In utero embryonic development
Anterior/posterior specification
Digestive tract development
Extracellular matrix organization
Head development
CNS neuron axonogenesis
Regulation of MAPK cascade
Neuron maturation
-log10 (P-value)
F
Bivalent gene
Pol II (+)
Pol II (-)
Percentage
100%
50%
0%
E3.5 ICM
E5.5 Epi
E6.5 Epi
E7.5 Mes
Normalized RPKM
Pol II
H3K4me3
H3K27me3
G
Gene expression of bivalent gene
Identified in E5.5 Epi
Identified in E6.5 Epi
FPKM
Pol II (+)
Pol II (-)
8.2e-5
3.7e-5
2.7e-9
3.3e-4
E5.5 Epi
E6.5 Epi
Ect
Mes
End
E8.5 Head
E9.5 Head
E14.5 Head
Cortex

Figure S3. Dynamic remodeling of Pol II (+) bivalent genes during germ layer formation. (**A**). Bar plots showing the number of bivalent genes identified in each germ layer and mESCs. (**B**). Venn diagram indicating overlap between bivalent genes at E6.5 and E7.5. (**C**). Sankey diagram illustrating the dynamic gain or loss of Pol II occupancy at bivalent promoters during transitions from E6.5 to E7.5 across germ layers. The “None” category includes genes lacking either bivalent chromatin features or detectable promoter-associated Pol II at the indicated stages. (**D**). Schematic illustrating developmental transitions of Pol II (+) bivalent genes across embryonic stages. Newly gained and lost genes are represented as elliptical nodes, with representative examples listed. Note that the endoderm originates from both the epiblast and the visceral endoderm. (**E**). GO terms associated with Pol II gain/loss gene clusters in panel (**D**). (**F**). Bar plots (left) quantifying Pol II (+) bivalent genes in E7.5 mesoderm and Pol II binding status in earlier stages (ICM, E5.5 Epi, E6.5 Epi). Box plots (right) showing enrichment of Pol II, H3K4me3, and H3K27me3 dynamics at both Pol II (+) and Pol II (–) bivalent promoters in the same gene sets. (**G**). RNA expression dynamics of Pol II (+) vs. Pol II (–) bivalent genes identified in E5.5 Epi (left) and E6.5 Epi (right). Their RNA levels in Ect, Mes, End, E8.5 Head, E9.5 Head, E14.5 Head and 8-week Cortex are also shown.

Figure S4

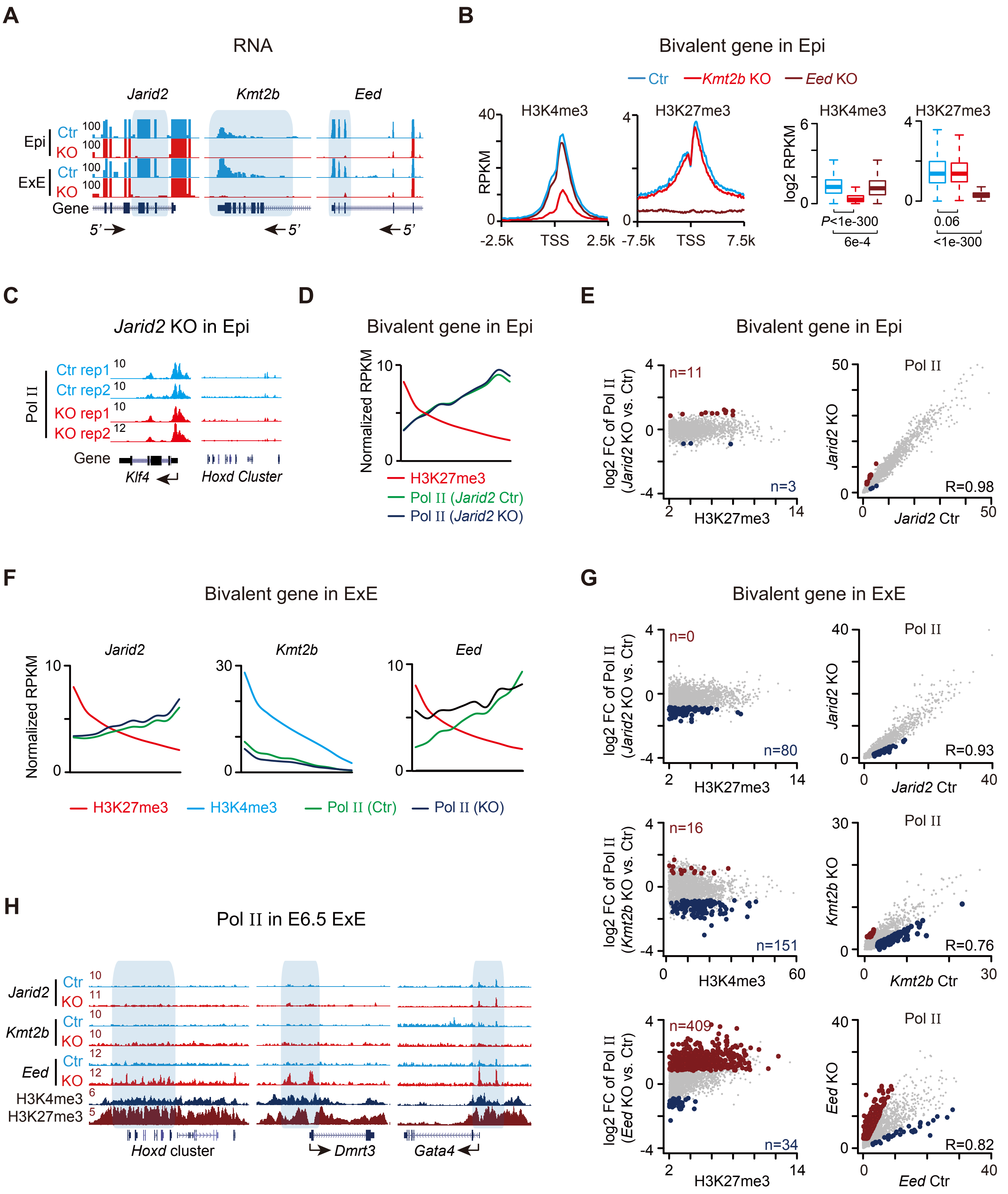

A
RNA
Jarid2
Kmt2b
Eed
Epi
ExE
Ctr
KO
100
Gene
5'
B
Bivalent gene in Epi
Ctr
Kmt2b KO
Eed KO
H3K4me3
H3K27me3
RPKM
log2 RPKM
TSS
-2.5k
2.5k
-7.5k
7.5k
P<1e-300
6e-4
0.06
<1e-300
C
Jarid2 KO in Epi
Pol II
Ctr rep1
Ctr rep2
KO rep1
KO rep2
Klf4
Hoxd Cluster
D
Bivalent gene in Epi
Normalized RPKM
H3K27me3
Pol II (Jarid2 Ctr)
Pol II (Jarid2 KO)
E
Bivalent gene in Epi
log2 FC of Pol II (Jarid2 KO vs. Ctr)
n=11
n=3
H3K27me3
Pol II
Jarid2 KO
Jarid2 Ctr
R=0.98
F
Bivalent gene in ExE
Normalized RPKM
H3K27me3
H3K4me3
Pol II (Ctr)
Pol II (KO)
G
Bivalent gene in ExE
n=0
n=80
R=0.93
log2 FC of Pol II (Kmt2b KO vs. Ctr)
n=16
n=151
H3K4me3
Kmt2b KO
Kmt2b Ctr
R=0.76
log2 FC of Pol II (Eed KO vs. Ctr)
n=409
n=34
Eed KO
Eed Ctr
R=0.82
H
Pol II in E6.5 ExE
Hoxd cluster
Dmrt3
Gata4

Figure S4. Histone modifications modulate Pol II recruitment at bivalent promoters. (**A**). Genome browser views of RNA-seq data in control and *Jarid2*, *Kmt2b*, and *Eed* knockout embryos in E6.5 Epi and ExE. Targeted deletion regions are highlighted. (**B**). Line plots display the enrichment of H3K4me3 and H3K27me3 in control and *Kmt2b-* or *Eed*-knockout Epi (left) at bivalent genes. Box plots (right) showing the signal intensity of the same gene sets. (**C**). The UCSC genome browser shows the Pol II occupancy at bivalent promoters in control and *Jarid2* knockout Epi. (**D**). Average plots ranking bivalent promoters in E6.5 epiblasts by H3K27me3 signal. Pol II signals from control and *Jarid2* knockout embryos are correspondingly shown. (**E**). Scatter plot (left) showing correlation between promoter H3K27me3 and fold change of Pol II enrichment in epiblast (*Jarid2* knockout vs. control). Red and blue dots indicate ≥2-fold up- or down-regulated genes, respectively. Scatter plot (right) comparing Pol II signals between control and *Jarid2* knockout embryos in epiblast. (**F**). Average plots ranking bivalent promoters in E6.5 ExE by H3K4me3 or H3K27me3 signal. Pol II signals from control and *Jarid2*, *Kmt2b* and *Eed* knockout embryos are correspondingly shown. (**G**). Scatter plot (left) showing correlation between promoter H3K27me3/H3K4me3 and fold change of Pol II enrichment in ExE (*Jarid2-*, *Kmt2b-*, or *Eed*-knockout vs. control). Red and blue dots indicate ≥2-fold up- or down-regulated genes, respectively. Scatter plot (right) comparing Pol II signals between control and these knockout embryos. (**H**). Genome browser views showing Pol II, H3K4me3, and H3K27me3 enrichment at bivalent promoters in control and gene knockout E6.5 ExE.

Figure S5

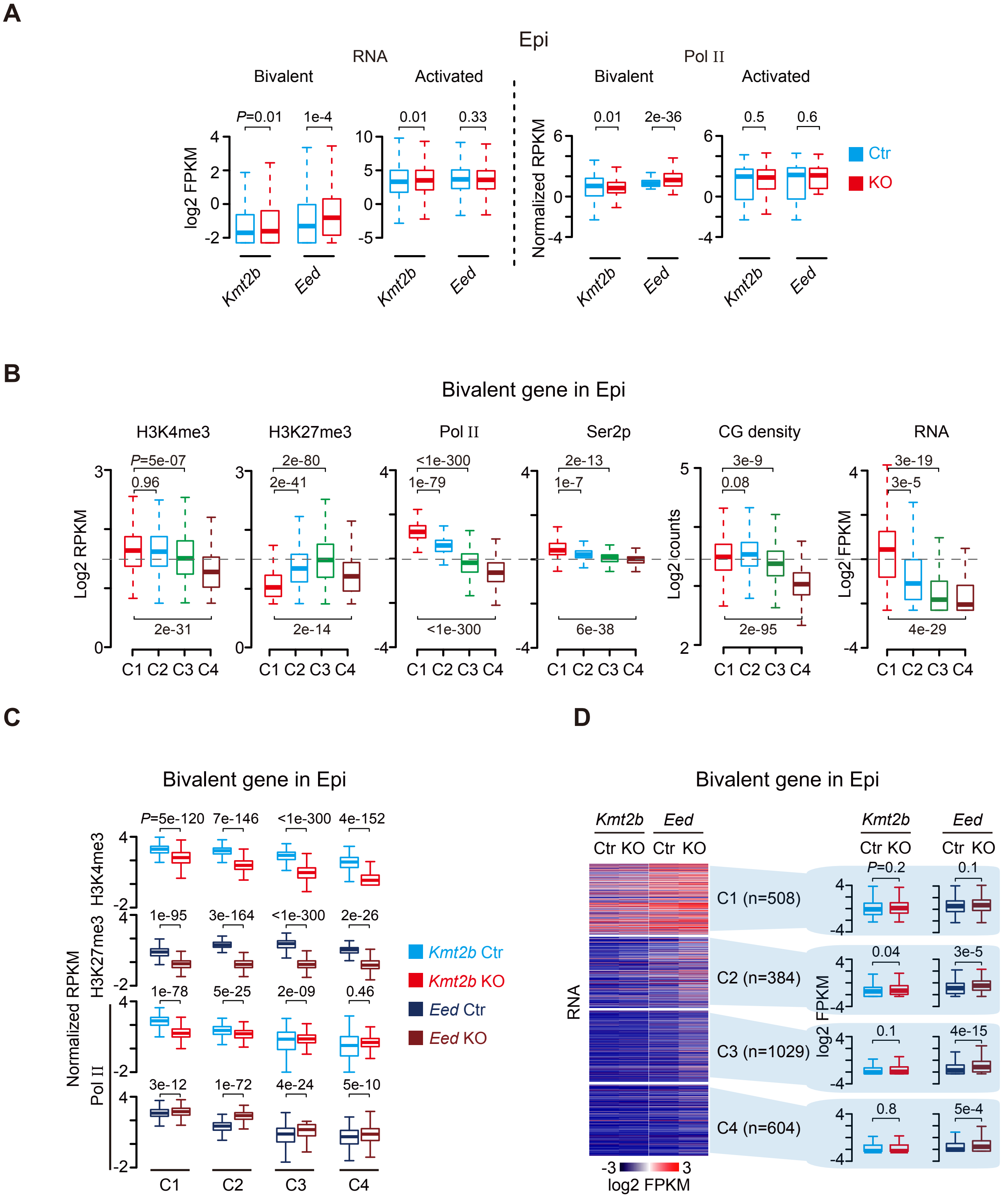
A
Epi
RNA
Pol II
Bivalent
Activated
Bivalent
Activated
log2 FPKM
Normalized RPKM
P=0.01
1e-4
0.01
0.33
0.01
2e-36
0.5
0.6
Kmt2b
Eed
Ctr
KO
B
Bivalent gene in Epi
H3K4me3
H3K27me3
Pol II
Ser2p
CG density
RNA
Log2 RPKM
Log2 counts
Log2 FPKM
P=5e-07
0.96
2e-31
2e-80
2e-41
2e-14
<1e-300
1e-79
<1e-300
2e-13
1e-7
6e-38
3e-9
0.08
2e-95
3e-19
3e-5
4e-29
C1 C2 C3 C4
C
Bivalent gene in Epi
Normalized RPKM
H3K4me3
H3K27me3
Pol II
P=5e-120
7e-146
<1e-300
4e-152
1e-95
3e-164
<1e-300
2e-26
1e-78
5e-25
2e-09
0.46
3e-12
1e-72
4e-24
5e-10
C1
C2
C3
C4
Kmt2b Ctr
Kmt2b KO
Eed Ctr
Eed KO
D
Bivalent gene in Epi
Kmt2b
Eed
Ctr KO
RNA
C1 (n=508)
C2 (n=384)
C3 (n=1029)
C4 (n=604)
log2 FPKM
P=0.2
0.1
0.04
3e-5
0.1
4e-15
0.8
5e-4

Figure S5. *Eed* deficiency leads to derepression of developmental genes. (**A**). Box plots comparing RNA expression (left) and Pol II (right) levels of bivalent and activated genes in control versus *Kmt2b*- and *Eed*-knockout Epi. (**B**). Box plots showing H3K4me3, H3K27me3, Pol II, Ser2p, CpG density, and RNA expression across four bivalent gene clusters (C1–C4). (**C**). Box plots showing H3K4me3, H3K27me3 and Pol II levels across four clusters (C1–C4) of bivalent promoters defined by chromatin signature. (**D**). Heatmaps showing RNA levels across four bivalent gene clusters (C1–C4) in *Kmt2b* and *Eed* knockout epiblasts. Gene numbers and box plot quantifications are shown on the right.

Figure S6

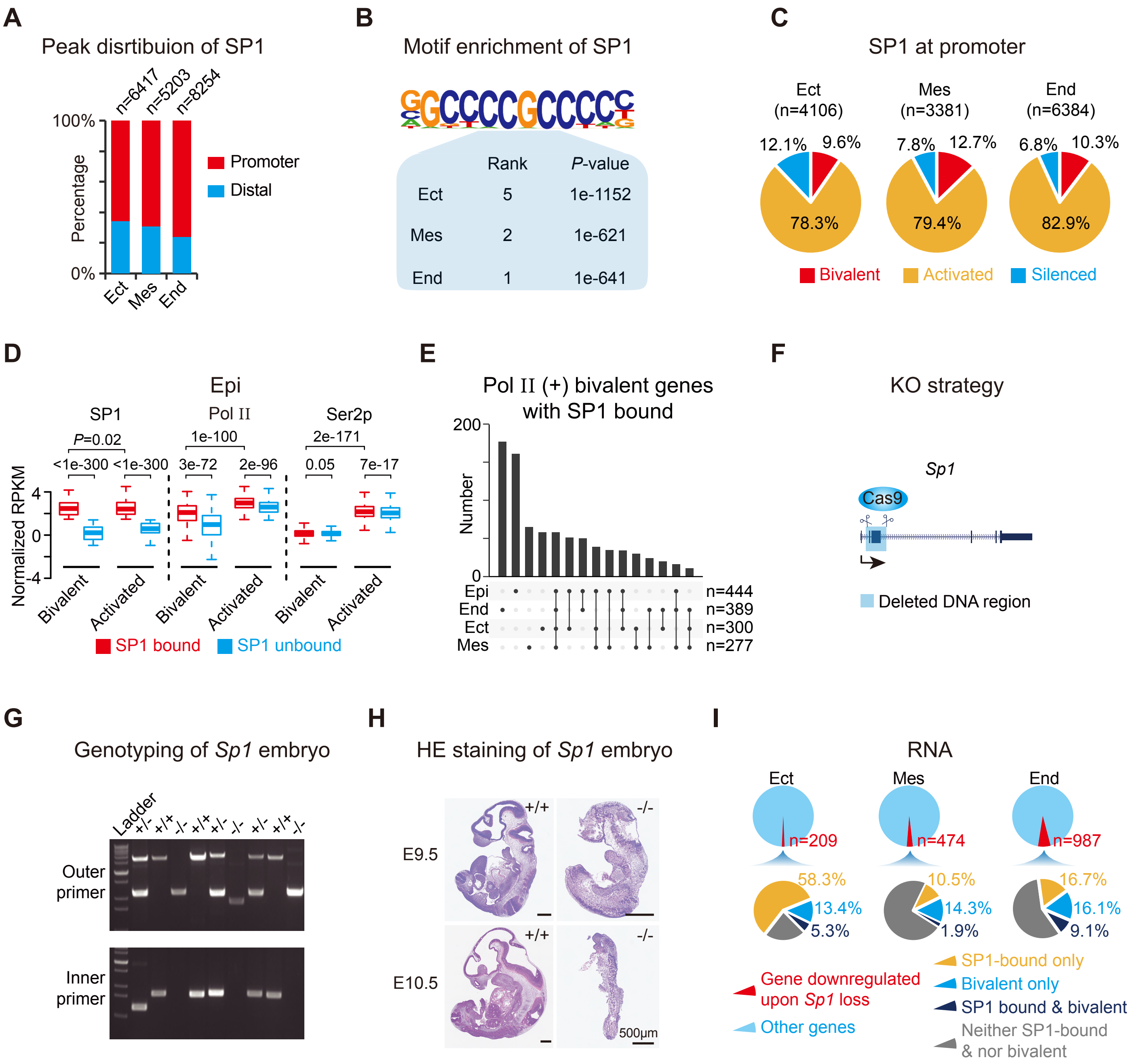

A
Peak disrtibuion of SP1
n=6417
n=5203
n=8254
100%
0%
Percentage
Promoter
Distal
Ect
Mes
End
B
Motif enrichment of SP1
Rank
P-value
Ect 5 1e-1152
Mes 2 1e-621
End 1 1e-641
C
SP1 at promoter
Ect (n=4106)
Mes (n=3381)
End (n=6384)
12.1% 9.6% 78.3%
7.8% 12.7% 79.4%
6.8% 10.3% 82.9%
Bivalent
Activated
Silenced
D
Epi
SP1
Pol II
Ser2p
P=0.02
1e-100
2e-171
<1e-300 <1e-300 3e-72 2e-96 0.05 7e-17
Normalized RPKM
Bivalent
Activated
SP1 bound
SP1 unbound
E
Pol II (+) bivalent genes with SP1 bound
Number
Epi n=444
End n=389
Ect n=300
Mes n=277
F
KO strategy
Sp1
Cas9
Deleted DNA region
G
Genotyping of Sp1 embryo
Ladder
Outer primer
Inner primer
H
HE staining of Sp1 embryo
E9.5
E10.5
+/+
-/-
500μm
I
RNA
Ect
Mes
End
n=209
n=474
n=987
58.3% 13.4% 5.3%
10.5% 14.3% 1.9%
16.7% 16.1% 9.1%
Gene downregulated upon Sp1 loss
Other genes
SP1-bound only
Bivalent only
SP1 bound & bivalent
Neither SP1-bound & nor bivalent

Figure S6. SP1 facilitates lineage specific Pol II recruitment at bivalent genes. (**A**). Bar plot showing SP1 peak distribution across genomic regions in each germ layer. (**B**). Ranked SP1 motif enrichment of SP1 peak identified in the three germ layers. (**C**). Pie charts showing the distribution of SP1-bound promoters among bivalent, activated and silenced genes across the three germ layers. (**D**). Box plots showing SP1, Pol II, and Ser2p levels in SP1-bound versus SP1-unbound subsets of bivalent and activated genes. (**E**). UpSet plot showing the overlap of SP1-bound Pol II (+) bivalent genes among Epi, Ect, Mes, and End. (**F**). Schematic of *Sp1* knockout strategy using CRISPR-Cas9, targeted exons are shaded and constitutively deleted. G. Genotyping of E7.5 *Sp1* knockout embryos. (**G**). Genotyping of E7.5 *Sp1* knockout embryos. (**H**). H&E stain of *Sp1* knockout embryos at E9.5 and E10.5. Scale bar: 500μm. (**I**). Pie charts showing the proportion of genes downregulated upon *Sp1* loss that are further classified as SP1-bound only, bivalent only, both SP1-bound and bivalent, or other genes in the three germ layers.

Figure S7

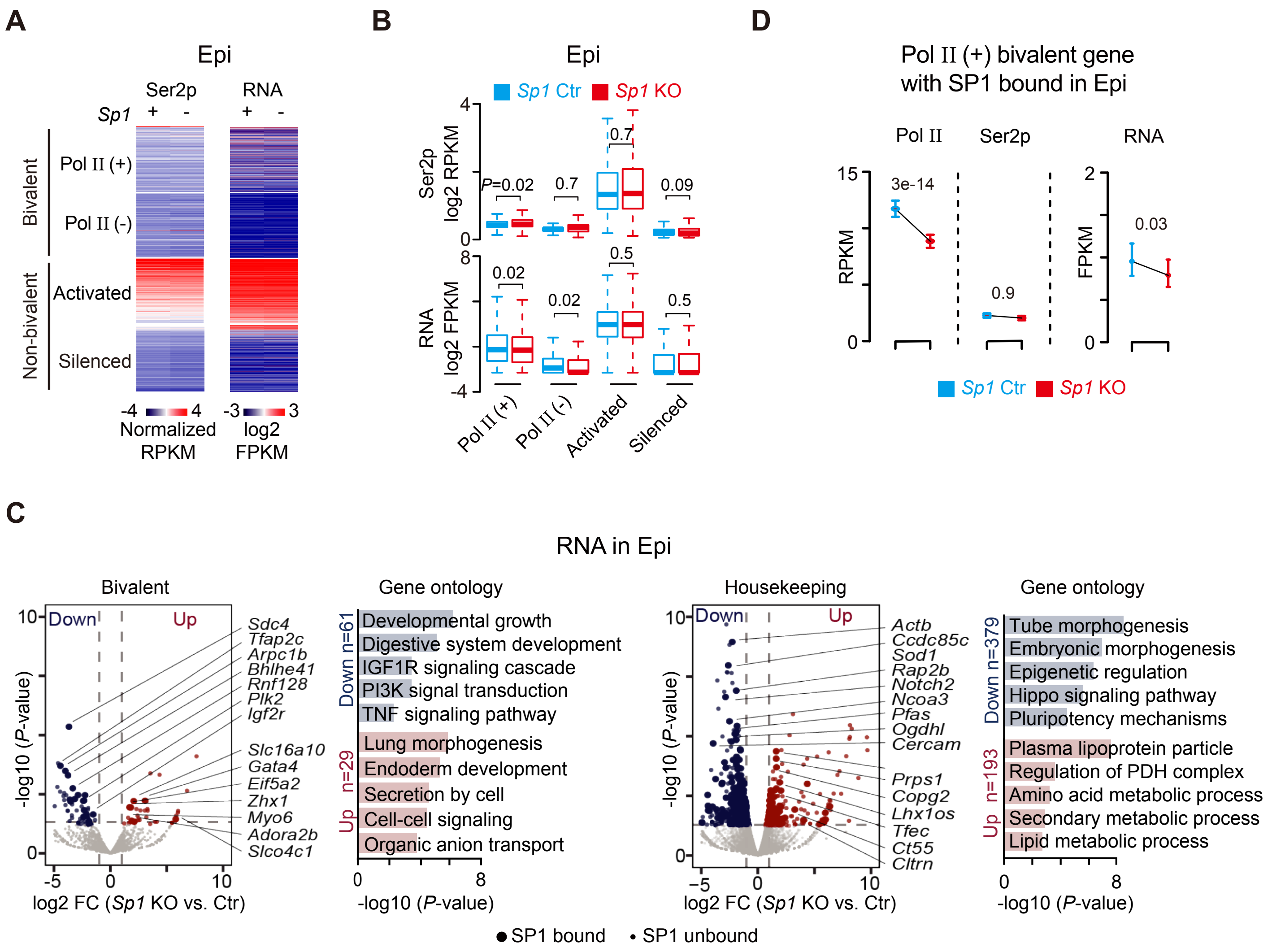

A
Epi
Ser2p
RNA
Sp1
+
-
Bivalent
Pol II (+)
Pol II (-)
Non-bivalent
Activated
Silenced
-4 4 Normalized RPKM
-3 3 log2 FPKM
B
Epi
Sp1 Ctr
Sp1 KO
Ser2p log2 RPKM
RNA log2 FPKM
P=0.02
0.7
0.09
0.02
0.5
Pol II (+)
Pol II (-)
Activated
Silenced
D
Pol II (+) bivalent gene with SP1 bound in Epi
Pol II
Ser2p
RNA
RPKM
FPKM
3e-14
0.9
0.03
C
RNA in Epi
Bivalent
Down
Up
-log10 (P-value)
log2 FC (Sp1 KO vs. Ctr)
Sdc4
Tfap2c
Arpc1b
Bhlhe41
Rnf128
Plk2
Igf2r
Slc16a10
Gata4
Eif5a2
Zhx1
Myo6
Adora2b
Slco4c1
Gene ontology
Down n=61
Developmental growth
Digestive system development
IGF1R signaling cascade
PI3K signal transduction
TNF signaling pathway
Up n=29
Lung morphogenesis
Endoderm development
Secretion by cell
Cell-cell signaling
Organic anion transport
-log10 (P-value)
Housekeeping
Actb
Ccdc85c
Sod1
Rap2b
Notch2
Ncoa3
Pfas
Ogdhl
Cercam
Prps1
Copg2
Lhx1os
Tfec
Ct55
Cltrn
Down n=379
Tube morphogenesis
Embryonic morphogenesis
Epigenetic regulation
Hippo signaling pathway
Pluripotency mechanisms
Up n=193
Plasma lipoprotein particle
Regulation of PDH complex
Amino acid metabolic process
Secondary metabolic process
Lipid metabolic process
SP1 bound
SP1 unbound

Figure S7. SP1 deficiency leads to aberrant gene expression. (**A**). Heatmaps showing Ser2p and RNA levels in *Sp1* knockout Epi across four gene sets: Pol Ⅱ (+), Pol Ⅱ (–), activated and silenced. (**B**). Box plots comparing Ser2p and RNA levels in *Sp1* knockout Epi using the same genes sets as shown in (**A**). (**C**). Volcano plots showing differential expression of bivalent (left) and housekeeping (right) genes in *Sp1* knockout versus control Epi. SP1-bound genes are highlighted; selected examples are annotated. Enriched GO terms for dysregulated genes are also shown. (**D**). Box plots showing Pol Ⅱ, Ser2p, and RNA levels at SP1-bound and SP1-unbound Pol Ⅱ (+) bivalent genes in control and *Sp1* knockout embryos.

Supplementary Tables

Supplementary tables were summarized in one Excel spreadsheet with:

Table S1. Sequences of sgRNA. (sheet 1)

Table S2. Primers for gene knockout embryo genotyping. (sheet 2)

Table S3. Lineage-specific genes identified by Ser2p across different germ layers. (sheet 3)

Table S4. Transcription decoupled genes across different germ layers. (sheet 4)

Table S5. Bivalent gene in different embryonic lineages. (sheet 5)

Table S6. Pol Ⅱ (+) bivalent gene in different embryonic lineages. (sheet 6)

Table S7. Sample information. (sheet 7)
